# Identifying Epithelial-Cell Progression Signatures (ECPSs) from Multi-Resolution Multi-Omics Data for Translational Clinical Applications in Lung Adenocarcinoma

**DOI:** 10.3390/ijms27156819

**Published:** 2026-07-29

**Authors:** Xueyao Chen, Tongxin Lv, Yang Wu, Fangfang Fan, Shaobo Kang, Renjie Dou, Wanmei Zhang, Dongxue Li, Rui Li, Yanyan Ping

**Affiliations:** College of Bioinformatics Science and Technology, Harbin Medical University, Harbin 150081, China; 2024020530@hrbmu.edu.cn (X.C.); 2019156034@hrbmu.edu.cn (T.L.); 2018156015@hrbmu.edu.cn (Y.W.); 2023020618@hrbmu.edu.cn (F.F.); 2021020504@hrbmu.edu.cn (S.K.); lazybean2021@163.com (R.D.); zwmei1998@gmail.com (W.Z.); 2023020569@hrbmu.edu.cn (D.L.); 18794244948@163.com (R.L.)

**Keywords:** epithelial-cell progression signature, multi-omics analysis, cancer-promoting signature, cancer-suppressing signature, lung adenocarcinoma, clinical application

## Abstract

Dynamic transcriptomic remodeling of epithelial cells represents a critical hallmark of lung adenocarcinoma (LUAD) progression, yet epithelial-cell progression signatures (ECPSs) linked to this process remain incompletely characterized, impeding our understanding of malignant LUAD transformation. Here, we performed a multi-resolution multi-omics study and identified two functionally opposing ECPSs: a Cancer-Promoting Signature (CPS) and a Cancer-Suppressing Signature (CSS). The CPS was progressively upregulated from normal to early- and advanced-stage lesions, while the CSS was gradually downregulated, and both were validated by multi-resolution transcriptomic data. Both the CPS and CSS demonstrated robust diagnostic value for LUAD, particularly in early-stage detection (median AUC > 0.95). In seven independent validation cohorts, the CPS and CSS served as robust prognostic risk and protective factors, respectively. Their combination could better stratify LUAD patients into distinct prognostic subgroups, with the CPS_high_–CSS_low_ subgroup showing the worst prognosis, accompanied by high genomic instability, an immunosuppressive tumor microenvironment, and resistance to chemotherapy. Notably, the CPS and CSS exhibited broad translational value in other epithelium-derived cancers. *HMGA1*, a key CPS gene, showed epithelium- and advanced-stage-specific high expression, which was significantly associated with poor prognosis, genomic instability, and immune escape. Collectively, our study identifies the CPS and CSS as clinically reliable ECPSs, providing valuable biomarkers for clinical application to LUAD.

## 1. Introduction

Lung adenocarcinoma (LUAD) is a lethal epithelial malignancy that is often diagnosed late, with aggressive progression and therapy resistance [1,2,3,4]. This is attributed to inadequate understanding of epithelial malignant transformation dynamics—specifically, the sequential, persistent transcriptional remodeling of epithelial cells throughout LUAD progression. As the cells of origin, epithelial cells undergo characteristic, persistent transcriptional remodeling during LUAD progression, acting as intrinsic drivers of malignancy [5]. Thus, deciphering these remodeling patterns and identifying epithelial-cell progression signatures (ECPSs) that reflect this dynamic process are critical to addressing clinical LUAD challenges.

Single-cell RNA sequencing (scRNA-seq) has revolutionized tumor heterogeneity research by resolving transcriptional shifts at cellular resolution and dissecting cell-type-specific dynamics previously masked in bulk transcriptomic analyses [6,7]. Several scRNA-seq studies have explored epithelial-cell dynamics in LUAD, providing preliminary insights. Sinjab et al. reported spatial heterogeneity of epithelial lineage between tumor and normal tissues using multi-regional scRNA-seq data of five early LUAD samples [8]. Single-cell networks revealed advanced LUAD cells have a more complex TME and higher ITH than early-stage cells [9]. Han et al. identified *KRT8*+ alveolar intermediate cells (KACs) as precancerous intermediates with reduced differentiation and increased plasticity [10]. *UBE2C*+ tumor cells drive LUAD invasion from adenocarcinoma in situ (AIS) through minimally invasive adenocarcinoma (MIA) to invasive adenocarcinoma (IAC) [11]. Zhang et al. identified the combination of *AQP1* and *AGTR2* as a hallmark of MIA’s basal-like phenotype via epithelial-cell analysis across normal, MIA and stage IA LUAD samples [12]. Despite these insights, most studies focus on discrete stages or specific cell states, failing to capture sequential transcriptional remodeling of epithelial cells across the entire progression spectrum. This limits our ability to link static molecular profiles to the dynamic continuum of malignant evolution. Thus, identifying intrinsic ECPSs of LUAD that encapsulate epithelial cells’ persistent transcriptional remodeling can bridge the gap between static molecular snapshots and the dynamic evolution of LUAD.

**Figure 1:**
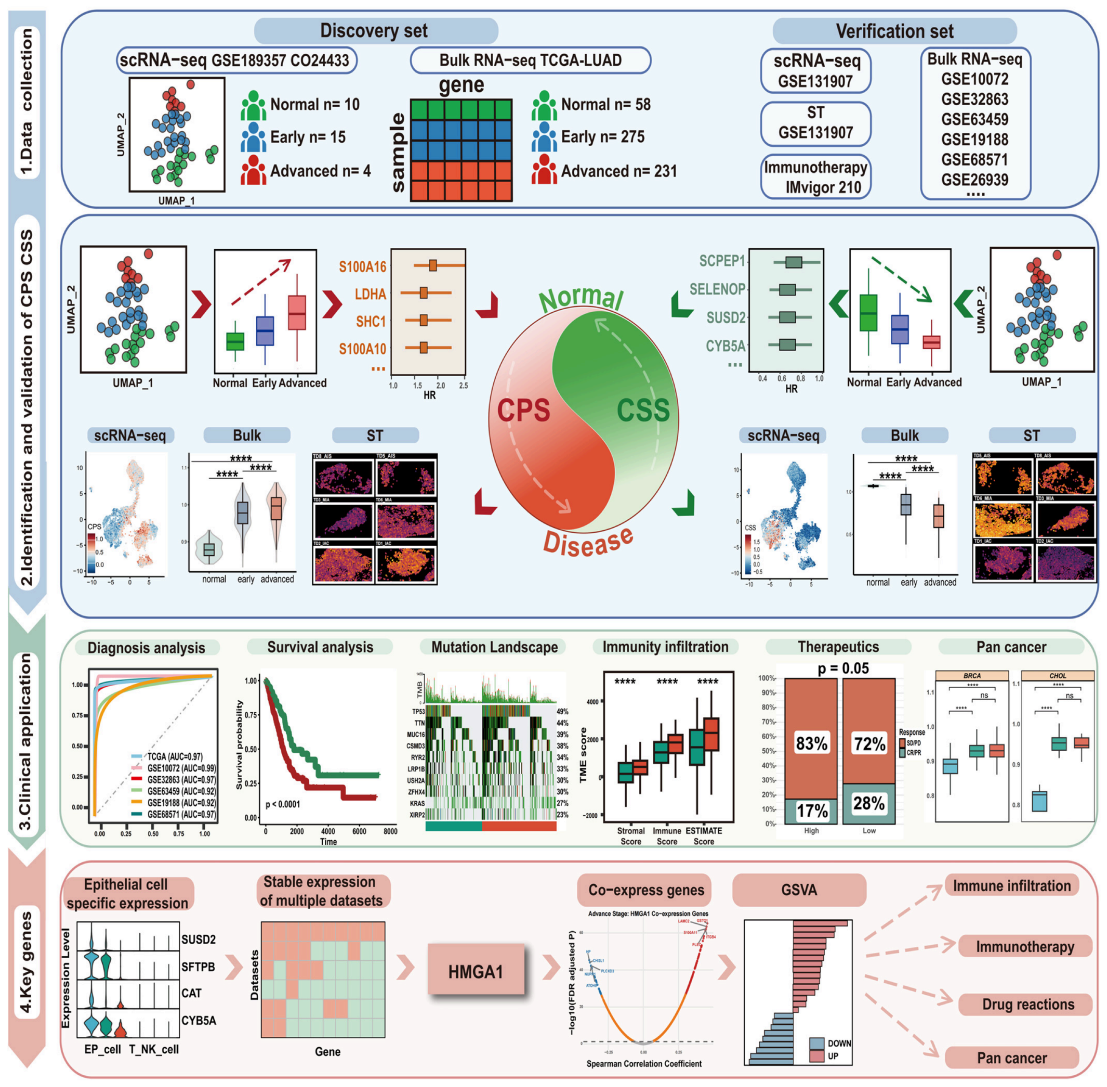
Workflow for the identification and validation of ECPSs. Significance: **** *p* < 0.0001 (Wilcoxon rank-sum test).

In this study, we investigated the transcriptomic remodeling of epithelial cells across the progression of LUAD, from normal tissue to early-stage and advanced-stage tumors. We systematically identified and validated two functionally distinct ECPSs (a Cancer-Promoting Signature (CPS) and a Cancer-Suppressing Signature (CSS)) based on their dynamic expression patterns and clinical relevance (Figure 1). These signatures were characterized by evaluating their diagnostic and prognostic utility, associations with genomic alterations, modulation of the tumor immune microenvironment, and implications for drug response. Additionally, we identified *HMGA1* as a key gene associated with LUAD progression and explored its potential as a biomarker and therapeutic target. Collectively, ECPSs can not only capture the sequential transcriptional remodeling of epithelial cells during LUAD progression but also hold considerable promise for clinical translation, laying a foundation for the translation of these signatures into LUAD precision medicine.

## 2. Results

### 2.1. Stage-Dependent Epithelial-Cell Remodeling in LUAD Points to Epithelial-Cell Progression Signatures (ECPSs)

LUAD is an epithelial cell-derived malignancy. To explore epithelial-cell remodeling trends during cancer progression, single-cell transcriptomic analysis was performed on 256,179 cells from 10 normal, 15 early-stage, and four advanced-stage samples in GSE189357 and CO24433. After data preprocessing and cell annotation, 28,242 epithelial cells were identified (Figure S1A–C). Unsupervised UMAP clustering of epithelial cells clearly distinguished three subgroups segregated by tumor stage (normal, early, and advanced), revealing striking transcriptional phenotypic heterogeneity across stages (Figure S2A). Epithelial-cell functional activity showed stage-specific trends: cilium movement, gas transport, and respiratory gaseous exchange progressively declined, while glucose metabolic processes, DNA replication, and cell-cycle G1/S and G2/M phase transitions significantly increased with disease progression (Figure S2B). Six hallmark cancer pathway activities in epithelial cells were significantly upregulated in a stage-dependent manner (Figure S2C). Collectively, these results demonstrate progressive epithelial phenotypic remodeling during LUAD development, providing critical insights for the identification of capturable epithelial-cell progression signatures. Specifically, we defined two opposing ECPS subsets associated with LUAD progression: a consistently upregulated CPS and a consistently downregulated CSS.

### 2.2. Identification and Multi-Resolution Validation of CPS During LUAD Progression

**Figure 2:**
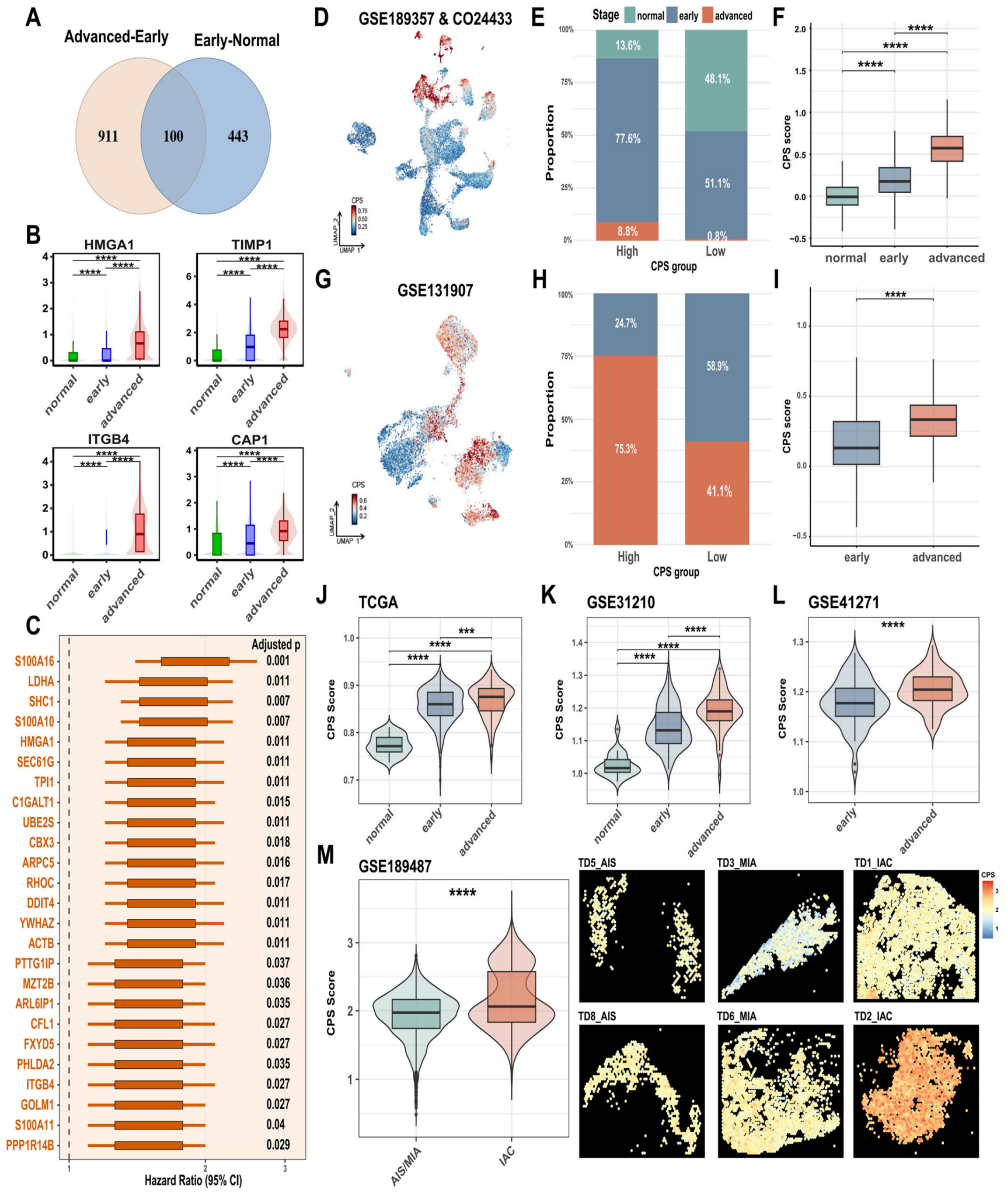
Identification and validation of the CPS. (**A**,**B**) Stage-dependent upregulated genes and their expression levels during LUAD progression. (**C**) Relationship between stage-dependent upregulation gene expression levels and OS assessed by univariate Cox survival analysis. (**D**–**I**) CPS scores across different stages in two scRNA-seq datasets. (**J**–**L**) CPS scores across different stages in three bulk RNA-seq cohorts. (**M**) CPS scores in AIS/MIA/IAC samples from spatial transcriptomic data. Significance: *** *p* < 0.001, **** *p* < 0.0001 (Wilcoxon rank-sum test).

We defined CPS genes based on two key features: (1) consistently up-regulated expression across the normal–early–advanced LUAD progression and (2) significant association between high expression and poor LUAD prognosis. A total of 100 persistently up-regulated genes were identified by comparing epithelial-cell transcriptomes across normal, early-stage, and advanced-stage LUAD samples (Figure 2A,B). Among these, high expression of 25 genes was significantly associated with poor prognosis (adjusted *p* < 0.05, Figure 2C). Several of these 25 genes have previously been reported to exert oncogenic effects in cancers. For example, *HMGA1* promotes LUAD progression by transcriptional activation of *DDAH1* in a *STAT1*-dependent manner [13] and can also activate the PI3K/AKT/mTOR pathway to enhance tumor-cell proliferation, migration, and invasion [14]. *S100A16* has been shown to promote cell proliferation, migration, invasion, and angiogenesis in LUAD [15]. These 25 genes constitute the CPS.

We performed multi-resolution validation of the CPS using single-cell, spatial, and bulk transcriptomes. In the discovery datasets (GSE189357 and CO24433), advanced-stage epithelial cells exhibited the highest CPS scores (Figure 2D). Compared with the CPS_low_ group, the CPS_high_ group showed higher proportions of early- and advanced-stage epithelial cells and lower proportions of normal epithelial cells (Figure 2E). CPS scores increased continuously and significantly from normal to early and advanced stages (Figure 2F). In the independent single-cell GSE131907 dataset (eight early-stage and seven advanced-stage LUAD samples), the CPS_high_ group contained a significantly higher proportion of advanced-stage epithelial cells (Figure 2G,H), and advanced-stage epithelial cells had significantly higher CPS scores than early-stage epithelial cells (Figure 2I). Bulk transcriptomic analysis of three cohorts (TCGA-LUAD, GSE31210, and GSE41271) confirmed stage-dependent CPS upregulation (Figure 2J–L). In the spatial transcriptome dataset (GSE189487), IAC stage had significantly higher CPS scores than AIS/MIA stages (Figure 2M). These multi-resolution validation results confirm the CPS as a robust, reproducible progression-indicative signature in LUAD.

### 2.3. Identification and Multi-Resolution Validation of CSS During LUAD Progression

**Figure 3:**
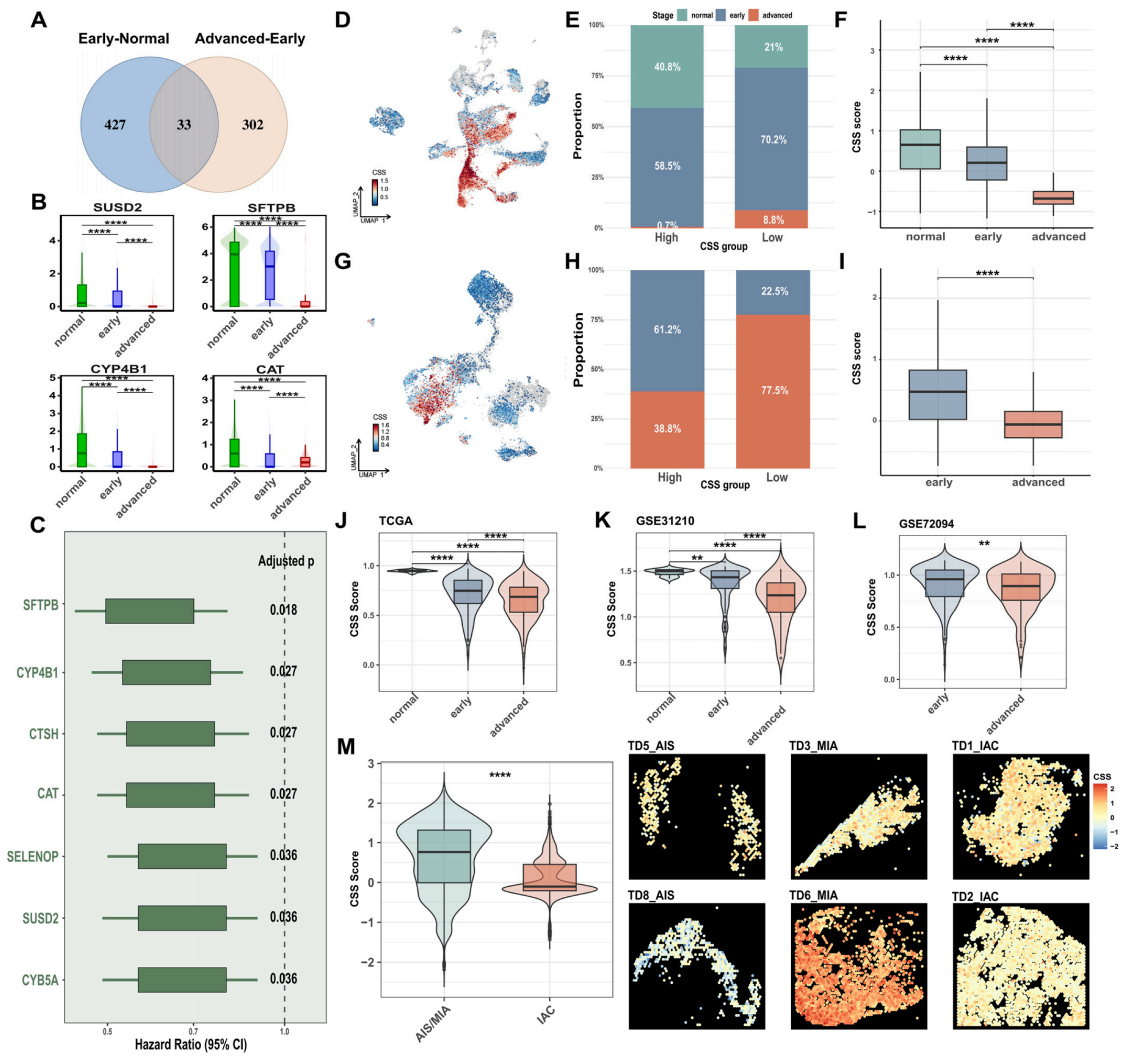
Identification and validation of the CSS. (**A**,**B**) Stage-dependent downregulated genes and their expression levels during LUAD progression. (**C**) Relationship between stage-dependent downregulation genes expression levels and OS assessed by univariate Cox survival analysis. (**D**–**I**) CSS scores across different stages in two scRNA-seq datasets. (**J**–**L**) CSS scores across different stages in three bulk RNA-seq cohorts. (**M**) CSS scores in AIS/MIA/IAC samples from spatial transcriptomic data. Significance: ** *p* < 0.01, **** *p* < 0.0001 (Wilcoxon rank-sum test).

We defined CSS genes based on two key features: (1) consistently down-regulated expression across the normal–early–advanced LUAD progression and (2) significant association between high expression and favorable prognosis in LUAD patients. A total of 32 persistently down-regulated genes were identified from normal to early- and advanced-stage LUAD (Figure 3A,B). Among these 32 down-regulated genes, seven genes were found to have high expression significantly associated with favorable prognosis (adjusted *p* < 0.05, Figure 3C). These seven genes (*SELENOP*, *SUSD2*, *CYB5A*, *CTSH*, *CAT*, *CYP4B1*, and *SFTPB*) formed the CSS. Among them, hypersialylation of *SUSD2* drives early LUAD progression by inducing ECM remodeling and activating pro-tumorigenic fibroblasts [16], consistent with a previously suggested protective role of unmodified *SUSD2* in LUAD. Pro-SFTPB, the precursor of *SFTPB*, has been reported to act as a tumor suppressor in NSCLC; its downregulation in primary NSCLC tissues correlates with high recurrence and poor prognosis in early-stage patients [17].

We also analyzed single-cell, spatial, and bulk transcriptomes to validate the CSS. In single-cell analysis, advanced-stage epithelial cells showed the lowest CSS scores (Figure 3D). Compared with the CSS_low_ group, the CSS_high_ group had a higher proportion of normal epithelial cells and lower proportions of early- and advanced-stage cells (Figure 3E). CSS scores continuously declined from normal to early- and advanced-stage cells (Figure 3F). Consistently, analysis of GSE131907 revealed significantly lower CSS scores in advanced-stage than early-stage epithelial cells (Figure 3G–I). Bulk transcriptome analyses (TCGA-LUAD, GSE31210, and GSE72094) confirmed stage-dependent CSS down-regulation (Figure 3J–L). Spatial transcriptomics (GSE189487) further showed significantly lower CSS scores in IAC than in AIS/MIA (Figure 3M). These findings establish the CSS as a reproducible progression-repressive signature in LUAD, functionally opposite to the CPS.

### 2.4. Dynamic Patterns of CPS and CSS During Malignant Epithelial Transformation

**Figure 4:**
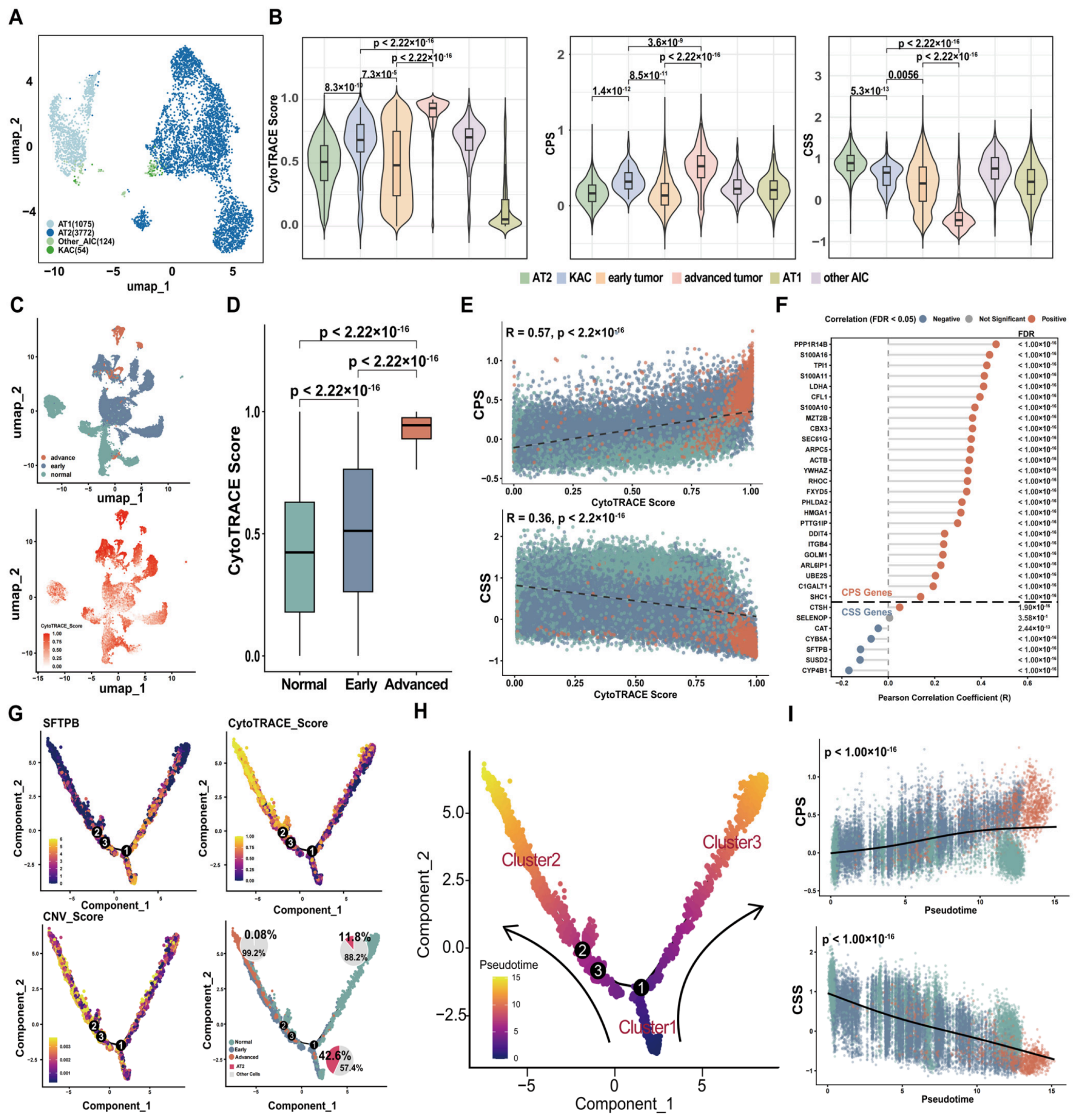
Association of the CPS and CSS with cellular stemness and malignant progression in LUAD epithelial cells. (**A**) UMAP of epithelial cells colored by cell type. (**B**) Violin plots showing CPS, CSS, and CytoTRACE scores across different epithelial cell types. (**C**,**D**) UMAP plots of epithelial cells colored by tumor stage and CytoTRACE score and box plot comparing CytoTRACE scores among normal, early-stage, and advanced-stage groups. (**E**,**F**) Correlations of CPS/CSS scores and signature genes with CytoTRACE scores. (**G**,**H**) Pseudotime developmental trajectory of epithelial cells, with Cluster 1 as the root based on higher *SFTPB* expression, lower CytoTRACE and CNV scores, and the highest AT2 cell proportion. (**I**) Dynamic changes of CPS and CSS scores along pseudotime.

To investigate how the CPS and CSS correlate with cellular states, cell differentiation potential and pseudotime trajectory, we first identified KAC cells as a transitional state among alveolar lineage cells using the annotation strategy of Han et al. [10] (Figure 4A and Figure S3). We found that KAC cells exhibited markedly higher CytoTRACE scores, higher CPS scores, and lower CSS scores relative to AT2 cells (Figure 4B). CytoTRACE scores increased progressively from normal to early and advanced malignant stages (Figure 4C,D). Correlation analysis showed that CPS scores exhibited significant positive correlation with CytoTRACE scores, while CSS scores exhibited significant negative correlation (Figure 4E,F). Furthermore, we constructed a pseudotime trajectory of epithelial-cell progression with Cluster 1 as the trajectory root. Cluster 1 exhibited higher SFTPB expression, lower CytoTRACE scores, a lower CNV burden and the highest proportion of AT2 cells (Figure 4G,H). Along pseudotime, CPS scores increased significantly, whereas CSS scores decreased significantly, with consistent trends for component genes (Figure 4I and Figure S4). These results demonstrate that epithelial cells with high CPS scores and low CSS scores may possess stronger stemness and higher malignant potential.

### 2.5. CPS and CSS as Robust Biomarkers for LUAD Diagnosis

**Figure 5:**
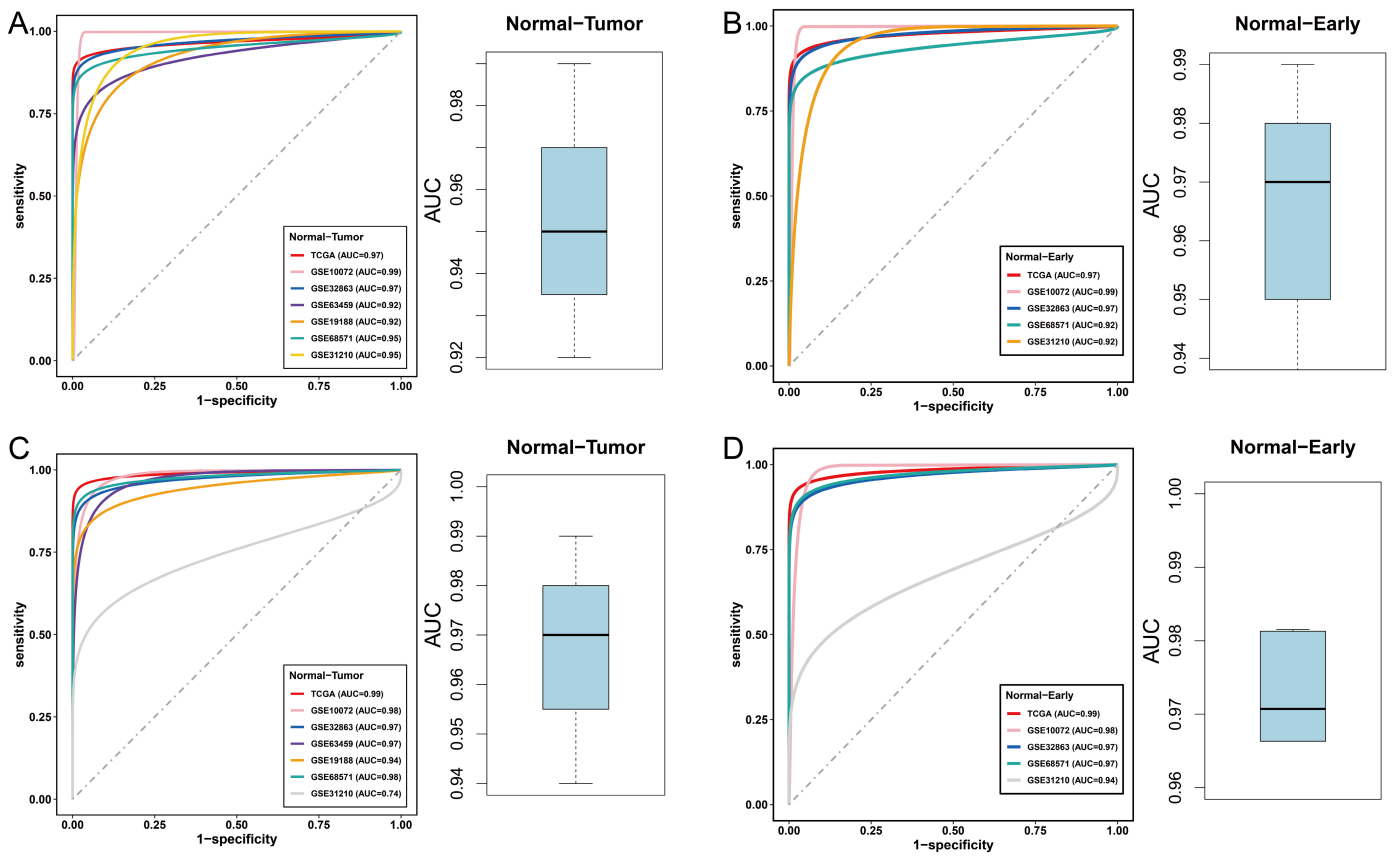
Diagnostic performance of the CPS and CSS in LUAD. (**A**) ROC curves and AUC distributions showing the diagnostic capability of the CPS in differentiating LUAD tumor from normal tissue. (**B**) ROC curves and AUC distributions showing the diagnostic capability of the CPS in differentiating early-stage LUAD tumors from normal tissue. (**C**) ROC curves and AUC distributions showing the diagnostic capability of the CSS in differentiating LUAD tumors from normal tissue. (**D**) ROC curves and AUC distributions showing the diagnostic capability of the CSS in differentiating early-stage LUAD tumors from normal tissue. The dashed diagonal line represents the reference line of random classification (AUC = 0.5).

We systematically validated the diagnostic utility of the CPS and CSS as robust biomarkers for LUAD through multi-cohort analysis. The median AUC of the CPS for distinguishing normal from malignant tissues exceeds 0.95 across seven datasets, confirming high sensitivity and specificity in diagnosis (Figure 5A). Notably, the CPS also exhibited robust diagnostic accuracy in early-stage LUAD, with all AUC values exceeding 0.97—underscoring its potential for pre-symptomatic detection (Figure 5B). Similarly, the CSS showed strong diagnostic performance in differentiating normal from LUAD samples, with a median AUC exceeding 0.97 across seven datasets (Figure 5C), and retained high discriminatory capacity in early-stage disease (Figure 5D). Collectively, these results validate the CPS and CSS as promising diagnosis biomarkers for LUAD, especially for identifying pre-invasive or early-stage tumors.

### 2.6. Robust Prognostic Values of the CPS and CSS with Opposite Roles in LUAD

**Figure 6:**
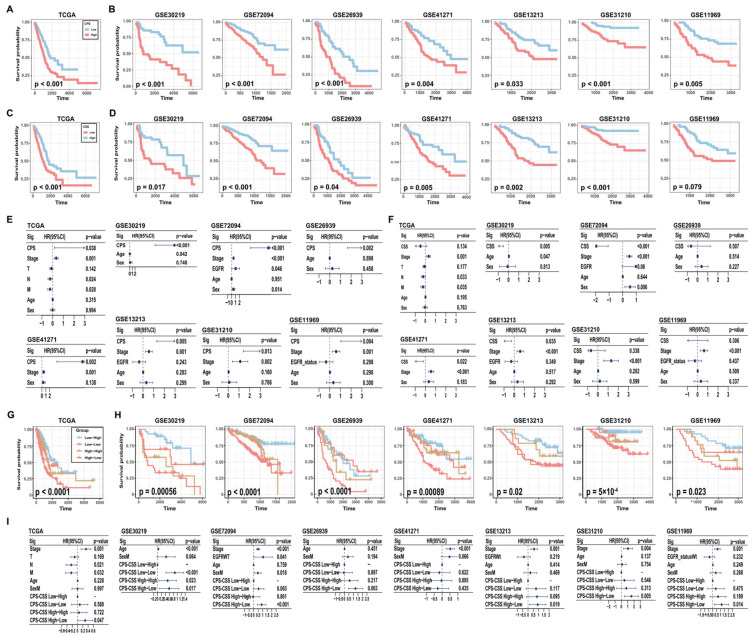
Prognostic value of the CPS and CSS and their combination in LUAD. (**A**) Survival analysis according to CPS stratification (median score) in TCGA-LUAD. (**B**) Survival analysis according to CPS stratification (median score) in the seven validation cohorts. (**C**) Survival analysis according to CSS stratification (median score) in TCGA-LUAD. (**D**) Survival analysis according to CSS stratification (median score) in the seven validation cohorts. (**E**) Multivariate Cox regression analyses for CPS. (**F**) Multivariate Cox regression analyses for CSS. (**G**) Survival analysis of patients stratified by the combined CPS and CSS in TCGA-LUAD. (**H**) Survival analysis of patients stratified by the combined CPS and CSS in the seven validation cohorts. (**I**) Multivariate Cox regression analyses for CPS–CSS groups.

The robust prognostic value of the CPS and CSS exhibiting opposite roles in LUAD was consistently validated in TCGA-LUAD and seven independent validation transcriptomic cohorts (Figure 6). For the CPS, LUAD samples were stratified into CPS_high_ and CPS_low_ groups based on the median score. Survival analysis revealed that CPS_high_ samples exhibited significantly shorter OS than CPS_low_ samples in both TCGA-LUAD and the seven validation cohorts (Figure 6A,B), with high CPS scores linked to adverse clinical outcomes, establishing the CPS as a robust prognostic risk factor for LUAD. Similarly, LUAD samples were stratified into CSS_high_ and CSS_low_ groups based on the median CSS score. CSS_high_ samples demonstrated significantly longer OS than CSS_low_ samples in both TCGA-LUAD and six of the seven validation cohorts (Figure 6C,D), confirming it as a reliable favorable prognostic marker. Moreover, the average time-dependent AUC values of the CPS and CSS for prediction of 1-, 3-, and 5-year survival all exceeded 0.65 across the seven independent datasets (Figure S5), further supporting their stable predictive efficacy. Multivariate Cox regression analyses showed that both CPS and CSS were significant and independent prognostic factors across multiple datasets (Figure 6E,F).

Given their opposite prognostic roles during LUAD progression, the CPS score was significantly negatively correlated with the CSS score in all datasets (Figure S6). To further explore their combined prognostic significance, we stratified LUAD samples into four groups: CPS_low_–CSS_high_, CPS_low_–CSS_low_, CPS_high_–CSS_high_, and CPS_high_–CSS_low_. Survival analysis showed significant, consistent survival differences among the four groups in both TCGA-LUAD and the seven independent validation cohorts (Figure 6G,H), with the most pronounced difference between the CPS_low_–CSS_high_ and CPS_high_–CSS_low_ groups. Multivariate Cox regression analyses showed that the CPS_high_–CSS_low_ group consistently exhibited the highest risk of poor prognosis (Figure 6I). To further translate these prognostic associations into a clinically applicable tool, we constructed a nomogram integrating the CPS, CSS, and clinical indicators (Figure S7A). The nomogram achieved 1-, 3-, and 5-year AUC values of 0.768, 0.758, and 0.707, respectively. Decision-curve analysis further verified that the nomogram yielded favorable net clinical benefit compared with the CPS alone, the CSS alone, and clinical variables used individually (Figure S7B).

### 2.7. High CPS/Low CSS Is Associated with Elevated TMB and Genomic Instability

The CPS was significantly positively correlated with TMB, whereas the CSS was significantly negatively correlated with TMB (Figure S8A). Significant TMB differences were observed across the four CPS- and CSS-stratified groups, with the CPS_low_–CSS_high_ group showing a significantly lower TMB than the CPS_high_–CSS_low_ group (Figure S8A). Groups stratified by CPS and CSS exhibited differential mutation frequencies among frequently mutated genes (Figure S8B). Significant mutation bias was identified in the recurrently mutated *TP53* gene, which displayed a significantly elevated mutation frequency in the CPS_high_, CSS_low_, and CPS_high_–CSS_low_ groups (Figure S8C). *TP53* plays a central role in maintaining genomic stability; its mutation frequency was elevated in CPS_high_ and CSS_low_ groups and highest in the CPS_high_–CSS_low_ subgroup (Figure S8D). In TCGA-LUAD and GSE72094 datasets, *TP53*-mutant tumors had significantly higher CPS scores and lower CSS scores than *TP53*-wild tumors (Figure S8E). Our findings reveal that a high CPS score or low CSS score is associated with elevated TMB and genomic instability in LUAD, highlighting the CPS and CSS as promising biomarkers.

### 2.8. High CPS/Low CSS Correlates with Changes in the Tumor Microenvironment, Enhanced Immune Escape, and Poor Immunotherapy Response

To explore the relationship between the CPS/CSS and tumor immune infiltration, tumor immune microenvironment components of TCGA-LUAD samples were analyzed using CIBERSORT, XCell and ESTIMATE. Correlation analysis showed that CPS and CSS scores were significantly associated with cellular enrichment and relative proportions in the tumor microenvironment (Figure S9A). For example, activated CD4+ memory T cells were significantly positively correlated with the CPS. ESTIMATE scores were significantly positively correlated with the CSS and significantly lower in the CSS_low_ group and CPS_high_–CSS_low_ group (Figure S9B). These results clearly demonstrate a close correlation between CPS/CSS dynamics and changes in the tumor microenvironment.

We then computed correlations between immune escape-associated pathways and either CPS or CSS scores. Our results revealed that Adhesion and Cytoskeleton pathway was positively correlated with CSS and exhibited the highest activity in the CSS_high_ group. In contrast, the Resistance to Cell Death and DNA Repair pathway was positively correlated with the CPS and displayed elevated activity in both the CPS_high_ group and the CPS_high_–CSS_low_ subgroup (Figure S9C,D). The TIDE algorithm was subsequently used to evaluate tumor immune-escape potential. Significantly higher TIDE scores were observed in the CPS_high_ and CSS_low_ groups, indicating greater tumor immune escape capacity in patients with high CPS scores and low CSS scores (Figure S9E).

To further explore the potential association between the CPS/CSS and immunotherapy response, we initially examined seven LUAD samples with immunotherapy outcome data from the GSE126044 cohort and observed lower CR/PR rates in both the CPS_high_ and CSS_low_ groups (Figure S9F). Given the limited sample size, we further expanded the exploratory analysis to the widely used IMvigor210 cohort. In IMvigor210, the CPS_high_ group exhibited lower CR/PR rates in response to immunotherapy (*p* = 0.01). Survival analysis showed poorer OS in the CPS_high_ group than the CPS_low_ group (*p* = 0.0016; Figure S9G). The CPS_high_–CSS_low_ group showed lower CR/PR rates (*p* = 0.05) and poorer OS (*p* = 0.00033; Figure S9H). These exploratory observations suggest that high CPS and low CSS scores were associated with remodeling of the tumor microenvironment and enhanced immune escape, which may provide potential clues for the prediction of immunotherapy response.

### 2.9. Association of the CPS/CSS with Anticancer Drug Sensitivity for Candidate Drug Screening in High-CPS/Low-CSS Patients

**Figure 7:**
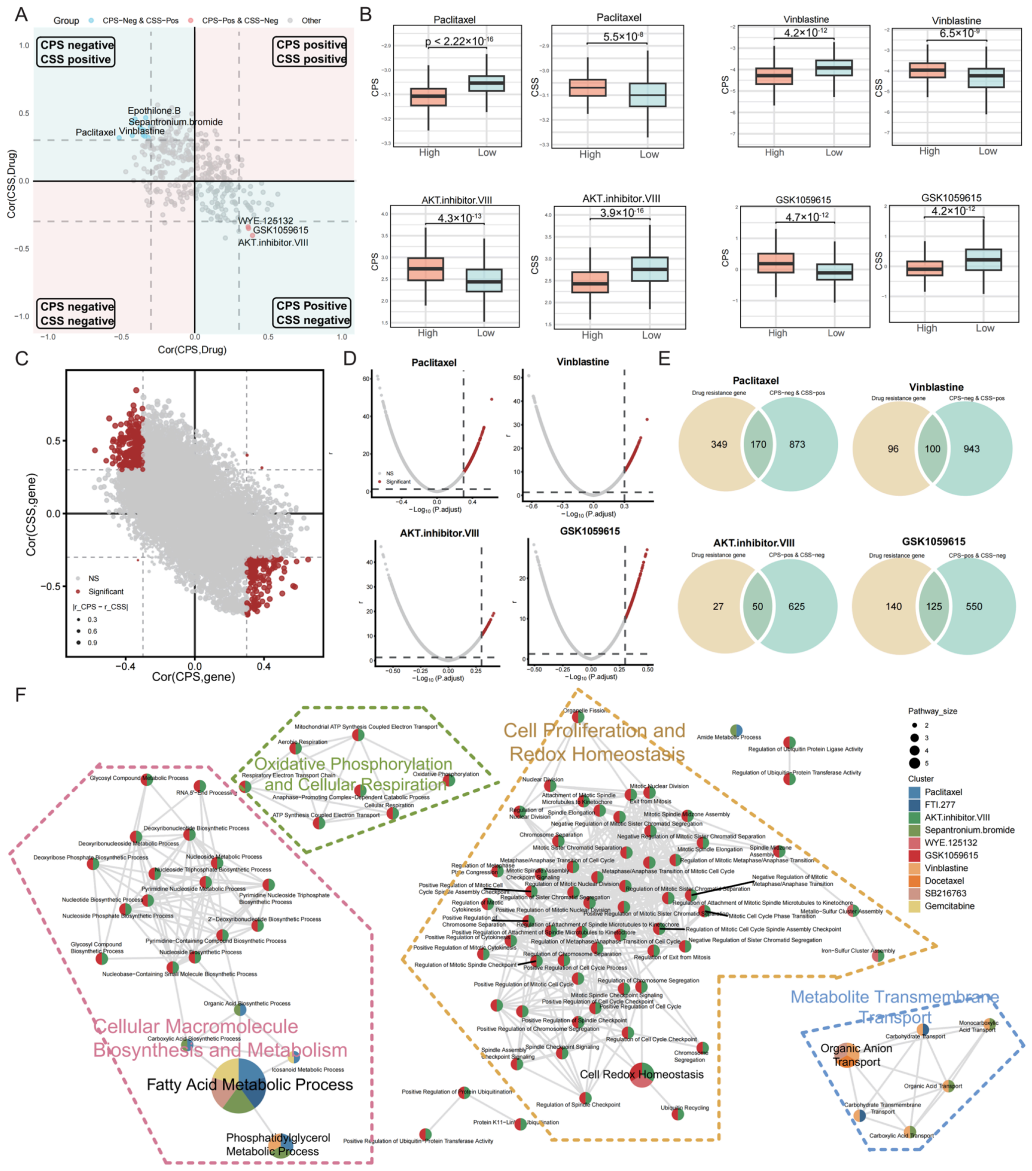
Association of the CPS/CSS with anticancer drug sensitivity in LUAD. (**A**) Correlation of CPS/CSS scores with 367 drug sensitivities (TCGA-LUAD). (**B**) Drug sensitivity comparisons with opposite CPS/CSS correlation patterns across CPS and CSS groups. (**C**–**E**) Identification of CPS/CSS-associated drug resistance genes for each of the 14 drugs. (**F**) Co-enrichment network of significantly enriched pathways across 14 drugs.

We predicted the sensitivity of 367 drugs in the TCGA-LUAD cohort to analyze the association between the CPS/CSS and anticancer drug sensitivity. Correlation analysis identified 14 drugs, including five conventional chemotherapeutic drugs (Paclitaxel, Vinblastine, Epothilone B, Docetaxel, and Gemcitabine) and nine targeted therapeutic drugs (AKT inhibitor VIII, GSK1059615, WYE-125132, FTI-277, sepantronium bromide, SB216763, CCT018159, rTRAIL, and AZD7762). The sensitivities of these drugs showed significantly opposite correlation patterns relative to the CPS and CSS (Figure 7A). Among these, AKT inhibitor VIII and GSK1059615 were positively correlated with the CPS but negatively correlated with the CSS (Figure 7A and Figure S10). Consistently, AKT inhibitor VIII and GSK1059615 exhibited significantly lower sensitivity in the CPS_low_ and CSS_high_ groups (Figure 7B), suggesting preferential efficacy in patients with low CPS and high CSS scores. Conversely, 11 drugs (including Paclitaxel and Vinblastine) were negatively correlated with the CPS but positively correlated with the CSS (Figure 7A and Figure S10). These drugs displayed significantly higher sensitivity in the CPS_high_ and CSS_low_ groups (Figure 7B), suggesting their therapeutic suitability for patients with high CPS and low CSS scores.

To further explore the molecular basis of CPS/CSS-associated drug resistance, for each of the 14 drugs, we identified potential resistance-associated genes that exhibited significantly positive correlations with drug sensitivity and also exhibited the same correlation patterns with the CPS/CSS as drug sensitivity (Figure 7C–E). Functional enrichment analysis was subsequently performed on these identified genes. The resistance genes associated with GSK1059615 were primarily enriched in pathways related to organelle fission and nuclear division (Figure S11). We further constructed a pathway network based on all significantly enriched functional pathways derived from the 14 drugs. The cell redox homeostasis pathway was enriched by the potential resistance genes associated with AKT inhibitor VIII, WYE.125132, and GSK1059615. The fatty acid metabolic process pathway was enriched by resistance genes from five drugs, including Paclitaxel and Vinblastine (Figure 7F and Figure S11). Collectively, these findings suggest that the CPS and CSS may serve as promising biomarkers to guide individualized anticancer drug selection for stratified LUAD patients.

### 2.10. CPS and CSS Demonstrate Broad Applicability Across Epithelial Malignancies

To evaluate the pan-cancer applicability of the CPS and CSS, their scores were computed in nine TCGA epithelial malignancy cohorts: BLCA, CHOL, COAD, ESCA, KIRP, LIHC, LUSC, STAD and THCA. Tumor samples showed significantly elevated CPS scores compared with normal tissues across all cancers (Figure S12A). The CPS exhibited exceptional diagnostic performance in CHOL (AUC = 1.00), LUSC (AUC = 0.98), and COAD (AUC = 0.96), with a median AUC exceeding 0.85 across all cancer types (Figure S12B). For early-stage tumors, the CPS maintained robust diagnostic accuracy, achieving AUC = 1.00 in CHOL and a median AUC of 0.85 (Figure S12C). In contrast, the CSS was significantly downregulated in tumor samples across most cancer types (Figure S12D). Diagnostic evaluation showed that the CSS achieved AUC = 0.99 for LUSC and 0.91 for KIRP and a median AUC exceeding 0.80 in distinguishing tumors from normal tissues (Figure S12E). For early-stage tumor diagnosis, the CSS also exhibited strong performance, with an AUC of 0.99 in LUSC and 0.92 in KIRP and a median AUC approaching 0.80 across all cancers (Figure S12F). Collectively, these results establish the CPS and CSS as robust diagnostic biomarkers across diverse epithelial malignancies, highlighting their broad applicability.

### 2.11. HMGA1 Is a Key CPS Gene Closely Associated with LUAD Progression with Epithelium and Advanced-Stage-Specific High Expression

**Figure 8:**
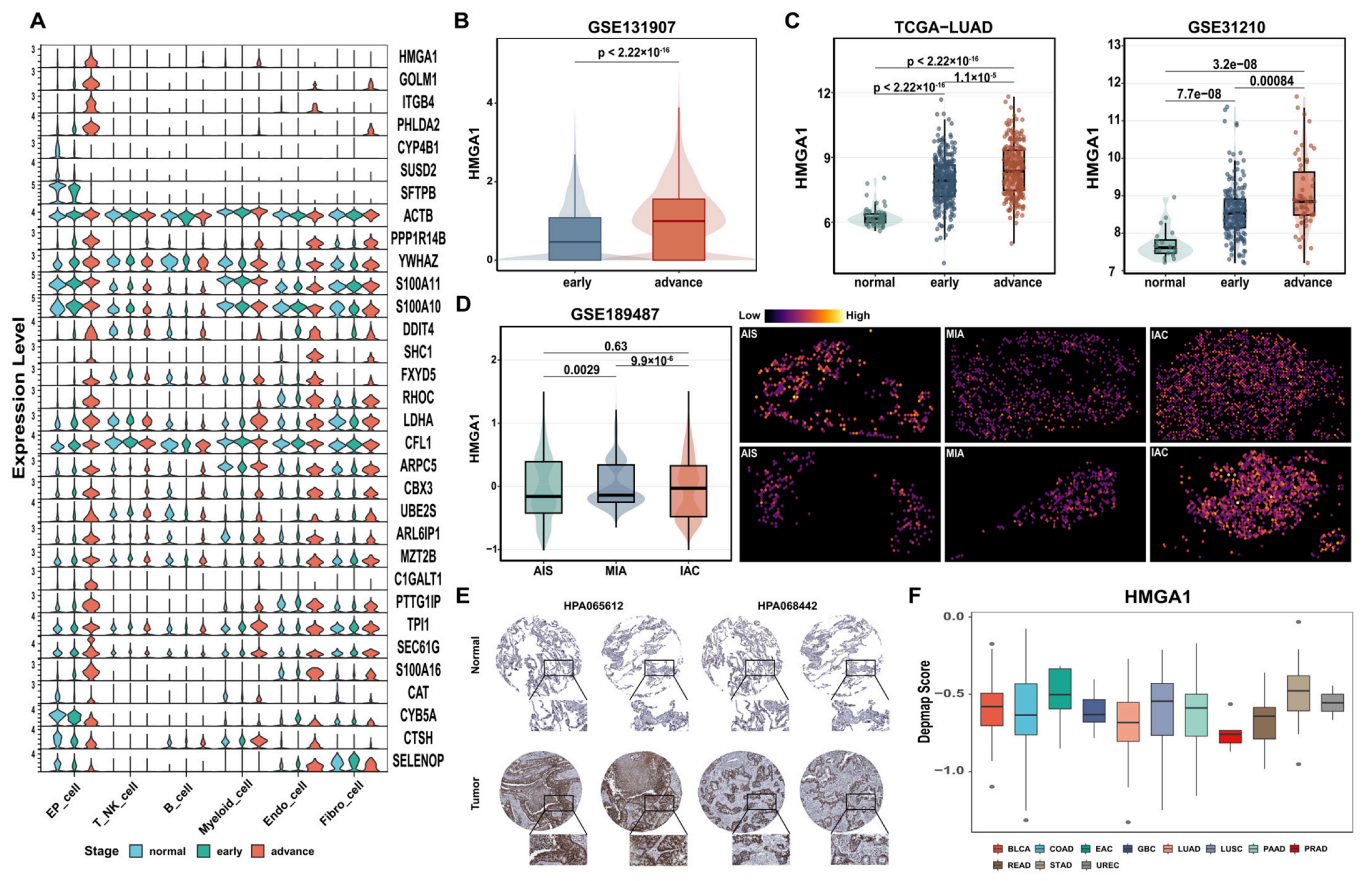
Identification of key genes associated with LUAD progression. (**A**) Expression levels of CPS and CSS genes in epithelial cells. (**B**–**D**) Expression level of *HMGA1* in scRNA-seq, spatial transcriptome, and bulk transcriptome. (**E**) IHC staining levels of *HMGA1* in various normal and tumor tissues. (**F**) *HMGA1* dependency on cancer-cell viability across multiple tumor types (DepMap). The black rectangular boxes indicate the regions shown at higher magnification in the corresponding insets below.

Seven CPS and CSS genes demonstrated epithelium-specific expression patterns: four CPS genes (*HMGA1*, *GOLM1*, *ITGB4*, and *PHLDA2*) exhibited advanced-stage-specific high expression in epithelial cells, while three CSS genes (*CYP4B1*, *SUSD2*, and *SFTPB*) were highly expressed in normal epithelial cells (Figure 8A). High *HMGA1* expression was consistently associated with poor prognosis, whereas SUSD2 and SFTPB acted as protective factors across multiple validation cohorts (Figure S13). Significant stage-dependent upregulation of *HMGA1* was validated by single-cell (GSE131907), spatial (GSE189487) and bulk transcriptome analyses (Figure 8B–D). Immunohistochemical (IHC) staining data of *HMGA1* in normal lung and LUAD tissues were obtained from the Human Protein Atlas (HPA) database using two independent antibodies (HPA065612 and HPA068442), revealing markedly higher *HMGA1* protein expression and stronger staining intensity in LUAD tissues than in matched normal lung tissues (Figure 8E). CRISPR data from DepMap showed that *HMGA1* knockout significantly impaired LUAD cell viability, with a median dependency score < −0.5 in most other epithelial malignancies (Figure 8F). Collectively, these findings support *HMGA1* as a critical oncogene candidate closely associated with LUAD progression, warranting further functional investigation.

To further characterize functional *HMGA1*, a co-expressed network of *HMGA1* was constructed in advanced-stage epithelial cells (*p* < 0.05, |r| > 0.3; Figure S14A,B). These genes were significantly enriched in cancer hallmarks, including KRAS_SIGNALING_UP, E2F_TARGETS, GLYCOLYSIS and HYPOXIA (Figure S14C). Multi-dataset validation demonstrated *HMGA1*’s robust diagnostic utility (AUC > 0.86) and risk role in LUAD prognosis (Figure S14D–I). TMB was significantly higher in the high-expression *HMGA1* group (Figure S14J). High *HMGA1* expression was linked to reduced immune-cell infiltration (Figure S14K). In the IMvigor210 cohort, patients with high *HMGA1* expression showed elevated TIDE scores and poorer survival (Figure S14L,M). *HMGA1* was also significantly upregulated in nine epithelial malignancies, with stage-dependent upregulation in ESCA, LIHC and KIRP (Figure S14N). Collectively, these findings highlight *HMGA1* as a critical candidate gene closely correlated with LUAD progression, with versatile oncogenic features suggested by previous literature and our multi-omics analyses.

## 3. Discussion

This study delineates transcriptional reprogramming of epithelial cells during LUAD progression, identifying two ECPSs (CPS and CSS) as robust biomarkers with diagnostic, prognostic, and therapeutic implications. Multi-omics validation demonstrated that CPS and CSS not only reflect dynamic evolution of LUAD but also are associated with genomic instability, remodeling of the immune microenvironment, and drug response. The antagonistic interplay between CPS and CSS reflects a yin–yang balance, highlighting their opposing roles in LUAD and filling a critical gap in understanding how opposing transcriptional programs are associated with LUAD progression. Furthermore, we identify *HMGA1* as a key oncogenic gene associated with epithelial plasticity, immune evasion, and pan-cancer relevance.

The CPS and CSS demonstrate multi-faceted clinical utility, validated through rigorous multi-cohort analyses. Their diagnostic power is exceptional, with median AUC = 0.95 for distinguishing tumor from normal tissues—even in early-stage detection—supporting risk stratification of indeterminate subsolid nodules. Prognostically, the CPS and CSS serve as independent biomarkers, with the CPS_high_-CSS_low_ subgroup exhibiting significantly worse survival outcomes. Therapeutically, these signatures may help inform precision interventions: CPS_high_ tumors are associated with enhanced sensitivity to Paclitaxel and Gemcitabine, while CSS_high_ cases are associated with a preferentially response to AKT inhibitor VIII. Notably, the observed potential correlation of high CPS scores with elevated TIDE scores and lower CR/PR rates may provide suggestive clues for interpreting immunotherapy resistance, yet reliable validation with larger dedicated LUAD immunotherapy cohorts is still required. Their pan-cancer applicability across nine epithelial malignancies underscores broad translational potential. Integrating the CPS and CSS may help clinicians to refine molecular risk stratification, optimize treatment selection, and personalize surveillance, advancing precision oncology in LUAD management.

The relationships between the CPS/CSS and mutation patterns, along with their associations, reveal intricate interactions among the genome, transcriptome, and immune microenvironment in LUAD. The CPS is positively correlated with TMB, while the CSS is negatively correlated with TMB, indicating that genomic mutations are associated with these signatures. Moreover, the CPS and CSS were associated with immune infiltration and distinct immune-cell populations—for example, the CPS_high_ group shows alterations in activated CD4+ memory T cells. These findings suggest that genomic changes reflected in mutation patterns are associated with transcriptional programs (CPS/CSS), which, in turn, are associated with the immune microenvironment, highlighting the complexity of LUAD progression.

We further explored the epigenetic regulation underlying CPS/CSS gene expression changes during LUAD progression using the scATAC-seq (GSE134812) data. Regulatory regions of CPS genes (including *HMGA1*) and CSS genes (including *CTSH*) exhibited significantly higher chromatin accessibility in malignant cells than in normal control cells (Figure S15). *HMGA1* harbored five significant accessible chromatin peaks in its regulatory regions. Two-sample Mendelian randomization analysis using multiple independent GWAS datasets (FinnGen, GWAS catalog and openGWAS) revealed that several CPS/CSS genes, including *CTSH*, *PPP1R14B*, *LDHA* and *CYP4B1*, were causally associated with LUAD risk. The consistency of *CTSH* and *PPP1R14B* across multiple cohorts, together with strong colocalization evidence for *CYP4B1*, further supports the robustness of the causal association (Figure S16). These findings, generated exclusively from public multi-omics datasets, offer preliminary insights into the epigenetic regulatory landscape and causal roles of CPS/CSS genes in LUAD.

Our study identifies *HMGA1* as a critical oncogenic candidate strongly correlated with LUAD progression. As a chromatin architectural protein, *HMGA1* has been reported to promote tumor progression by activating the PI3K/AKT signaling axis, enhancing aerobic glycolysis, and upregulating key glycolytic enzymes [18,19], ultimately impairing patient survival. This pro-survival function is supported by in vitro and in vivo evidence, including suppressed tumor growth in xenograft models upon *HMGA1* knockdown [18,20]. Additionally, *HMGA1* was associated with genomic instability and immune escape in our analyses, consistent with its oncogenic roles. Beyond *HMGA1*, we investigated CSS genes *SFTPB* and *SUSD2*, which exhibit strong diagnostic and early diagnostic potential for LUAD. High expression of these genes correlates with better prognosis (Figures S17 and S18), consistent with prior studies showing low *SFTPB*/*SUSD2* expression predicts worse outcomes [21,22,23]. Both genes are also associated with immune-cell infiltration, aligning with evidence that SFTPB modulates the TME—particularly in fostering an immunosuppressive environment [21]. Notably, the low-expression group exhibits lower TME scores but higher TMB and tumor purity (Figure S19), suggesting loss of SFTPB’s tumor-suppressive function—such as its inhibition of sPLA2 activity and arachidonic acid production in NSCLC [24]—may be associated with aggressive tumor behavior.

Beyond these well-characterized genes in the CPS/CSS, several other genes also warrant attention. *PHLDA2* (Pleckstrin homology-like domain, family A, member 2; also known as *IPL*/*TSSC3*) is involved in apoptosis, migration and autophagy and is associated with the development and progression of LIHC, BRCA and colorectal cancer (CRC) [25]. However, *PHLDA2* studies in LUAD remain limited; some show it is highly expressed in LUAD (mainly localized in epithelial cells and T cells) and promotes progression by regulating LUAD cell proliferation and migration [26], consistent with our findings. Additionally, *FXYD5* (Dysadherin), a cancer-associated cell-membrane glycoprotein that modulates pump activity to adapt to the cellular environment, is highly expressed in gastric, colorectal, pancreatic and breast tumors but low in normal tissues. Previous studies have indicated that high *FXYD5* expression promotes tumor metastasis and correlates with poor prognosis in breast cancer [27], while its role in LUAD and lung cancer remains largely unclear. Existing evidence has shown that upregulated *FXYD5* enhances chemokine *CCL2* expression and secretion, thereby promoting tumor growth, angiogenesis and metastasis; these findings are consistent with our observation that *FXYD5* is associated with oncogenic phenotypes in LUAD. Nevertheless, the specific mechanisms underlying these genes in the malignant LUAD progression remain poorly defined and require further investigation.

We further compared our CPS and CSS with six LUAD progression-associated signatures published in the past five years [28,29,30,31,32,33]. No overlapping genes were identified between our signatures and these published sets (Figure S20A). We then systematically evaluated their performance in both stage discrimination and prognostic stratification across multiple independent cohorts. For stage discrimination, we evaluated the ability of each signature to reflect disease progression in the TCGA-LUAD and GSE31210 cohorts. Only the signature from PMC12191159 [29], similar to our CSS, exhibited a continuous decline as the stages progressed in both TCGA-LUAD and GSE31210, whereas the other signatures showed no significant differences between early- and advanced-stage tumors (Figure S20B,C). For prognostic stratification, these six published signatures showed favorable performance only in a few datasets (Figure S20D). Given the advantages of our CPS and CSS in offering bidirectional roles, they may serve as valuable biomarkers for the evaluation of disease progression and prognosis.

While this study enhances reliability through multi-omics validation, some constraints remain. The single-cell analysis, which included only four advanced-stage LUAD cases (*n* = 4), may not fully capture the molecular diversity of advanced disease, given the well-recognized intertumoral heterogeneity. Although we performed extensive validation across bulk transcriptomic and spatial transcriptomic datasets, with consistent results supporting the robustness of our signatures, we acknowledge that a larger scRNA-seq cohort would be necessary to comprehensively address this limitation. Additional in vitro/in vivo functional studies are needed to elucidate the mechanistic roles of the CPS and CSS and further strengthen their clinical applicability.

## 4. Materials and Methods

### 4.1. Identification and Validation of Epithelial-Cell Progression Signatures (ECPSs)

#### 4.1.1. Definition of ECPSs

Epithelial-cell progression signatures (ECPSs) were defined as gene sets that are consistently upregulated or downregulated during the malignant progression of epithelial cells (from normal to early- and advanced-stage states) and whose high or low expression is significantly associated with poor prognosis. Based on their expression trends, ECPSs were categorized into two subgroups: the CPS and CSS. Specifically, the CPS refers to a set of genes that are consistently upregulated during the malignant progression of epithelial cells (from normal to early- and advanced-stage states) and whose high expression is significantly associated with poor prognosis. In contrast, the CSS represents a complementary set of genes that are consistently downregulated during epithelial-cell malignant progression and whose high expression is significantly linked to improved patient prognosis.

#### 4.1.2. Discovery Datasets and Preprocessing

The discovery datasets for ECPSs included two scRNA-seq datasets—GSE189357 (from the Gene Expression Omnibus, GEO; https://www.ncbi.nlm.nih.gov/geo/ (accessed on 17 July 2026))) [11] and CO24433 (from Code Ocean; https://codeocean.com/ (accessed on 17 July 2026))) [34]—as well as bulk RNA-seq and clinical survival data of LUAD patients from The Cancer Genome Atlas (TCGA; https://portal.gdc.cancer.gov/ (accessed on 17 July 2026))).

The scRNA-seq datasets (GSE189357 and CO24433) were processed using the Seurat package (v4.4.0) [35]. Quality-control measures included: (1) exclusion of genes present in fewer than three cells, (2) removal of cells with mitochondrial (mt) gene expression greater than 20% and hemoglobin (HB) gene expression greater than 10%, (3) removal of cells with fewer than 500 total RNA reads, and (4) removal of cells expressing fewer than 200 or more than 10,000 genes. Data normalization was performed using the LogNormalize method via the NormalizeData function, followed by scaling of the normalized expression matrix via the ScaleData function, while the top 2000 highly variable genes (HVGs) were identified via the FindVariableFeatures function. Batch effects were corrected using the Harmony method (v1.2.1) [36] to combine Seurat objects into a unified dataset. Dimensionality reduction was conducted via Principal Component Analysis (PCA), and cell clustering was executed using the FindNeighbors (dims = 1:25) and FindClusters (dims = 1:25, resolution = 0.5) functions. For visualization, the Uniform Manifold Approximation and Projection (UMAP) algorithm was applied. Cell types were initially inferred using the SingleR package (v2.6.0) [37,38] based on the GSE130148 dataset, while the annotation results were further revised and validated based on a literature review and canonical marker genes (e.g., EPCAM, MUC1, and KRT19 for epithelial cells). Finally, after filtering non-epithelial contaminants, we defined tumor/normal epithelial-cell clusters according to their original sample source, and tumor cluster reliability was verified via the InferCNV algorithm [39].

#### 4.1.3. Identifying ECPS Genes

Based on clinical sample staging, epithelial cells were stratified into three groups: normal, early-stage (MIA, AIS, IAC, and Stage I), and advanced-stage (Stages II–III). Differentially expressed genes (DEGs) across these three groups were identified with the FindMarkers function. The thresholds applied for DEG identification were |log_2_FC| > 0.25, adjusted *p*-value < 0.05, and expression ≥ 25% of cells in at least one group.

Univariate Cox proportional hazards regression was further performed to identify prognosis-associated genes from DEGs based on TCGA-LUAD-cohort and clinical survival data. Genes with adjusted *p* < 0.05 and a hazard ratio (HR) > 1 or <1 were retained. Those satisfying both the DEG screening and univariate Cox regression criteria were defined as ECPS genes.

#### 4.1.4. Validation of ECPSs

To validate the stability and reproducibility of CPS and CSS expression-change trends during LUAD progression, independent ECPS validation datasets were collected across three resolution levels: an scRNA-seq dataset (GSE131907), a spatial transcriptomic dataset (GSE189487), and four bulk RNA-seq datasets (TCGA-LUAD, GSE31210, GSE41271, and GSE72094).

For scRNA-seq data, pre-annotated epithelial-cell clusters were retrieved, and the AddModuleScore method was used to calculate CPS/CSS scores per cell for quantification of epithelial-cell malignancy. For bulk RNA-seq data, single-sample gene-set enrichment analysis (ssGSEA (v1.52.3)) [40] was applied to compute CPS/CSS scores per sample. For the spatial transcriptomic dataset (GSE189487), sample regions were annotated based on hematoxylin–eosin (HE) staining and classified into four histological categories: cancer, normal epithelial, stromal, and lymphoid regions. Standard preprocessing procedures were performed using the Seurat package, including data normalization, dimensionality reduction, clustering, and identification of spatially variable features. Spots with fewer than 200 detected genes or a mitochondrial transcript ratio > 20% were removed, and genes expressed in fewer than two spots were excluded. The count matrix was normalized using the SCTransform function. Cancer-region spots were extracted for downstream analysis, and CPS/CSS scores were calculated using the same AddModuleScore method as in the scRNA-seq analysis.

The CytoTRACE package (v0.3.3) was applied to evaluate the differentiation stemness of epithelial cells [41]. Pseudotime trajectory analysis was performed using Monocle2 (v2.10.0) [42] to reconstruct the evolutionary lineage of epithelial cells. The DDRTree algorithm was used for dimensionality reduction and cell ordering.

### 4.2. Analysis of the Clinical Value of ECPSs

#### 4.2.1. Evaluation of the Diagnostic Efficacy of ECPSs

To assess the diagnostic value of the CPS and CSS for LUAD, particularly for early-stage LUAD, eight bulk RNA-seq datasets (TCGA-LUAD, GSE10072, GSE11969, GSE32863, GSE63459, GSE19188, GSE68571, and GSE31210) with normal and LUAD samples were collected. CPS and CSS scores for each sample were calculated via ssGSEA (v1.52.3). Receiver operating characteristic (ROC) curve analysis was conducted using the pROC package (v1.18.5), with area under the curve (AUC) values calculated to quantify the diagnostic performance of the CPS and CSS.

#### 4.2.2. Evaluation of the Prognostic Value of ECPSs

To assess the prognostic value of the CPS and CSS for LUAD, TCGA-LUAD and the seven independent validation datasets (GSE72094, GSE41271, GSE31210, GSE30219, GSE26939, GSE13213, and GSE11969), transcriptomic and clinical survival data were collected. Based on the median CPS and CSS scores, samples were divided into high- and low-score groups. Kaplan–Meier survival curves were plotted, and log-rank tests were performed to compare overall survival (OS). Multivariate Cox regression analyses assessed the independent prognostic significance of the CPS and CSS. A nomogram integrating the CPS, CSS, and clinical variables was constructed using the rms package to predict individual survival probabilities. The discriminatory power and accuracy of the nomogram were validated using ROC curves, C-indices and calibration plots, and decision curve analysis (DCA) evaluated the clinical utility of the model.

#### 4.2.3. Correlation Analysis Between ECPSs and Tumor Mutation Patterns

To explore the association of the CPS and CSS with genomic alterations in LUAD, somatic mutation data of TCGA-LUAD were obtained. Tumor mutation burden (TMB) was calculated for each sample using the maftools package (v2.0.0) [43] to characterize the mutational landscape of LUAD samples.

#### 4.2.4. Correlation Analysis Between ECPSs and the Tumor Immune Microenvironment

Immune-cell infiltration levels of the LUAD samples were quantified using the CIBERSORT and XCell algorithms [44,45]. Spearman’s rank correlation was performed to evaluate the associations between CPS/CSS scores and the relative abundances of infiltrating immune-cell subsets. Additionally, the ESTIMATE algorithm [46] was applied to calculate immune, stromal, and tumor purity scores to characterize the composition of the tumor microenvironment (TME).

#### 4.2.5. Correlation Analysis Between ECPSs and Immunotherapy Efficacy

We calculated Tumor Immune Dysfunction and Rejection (TIDE) scores for TCGA-LUAD samples to assess tumor immune-evasion potential using the TIDE website (http://tide.dfci.harvard.edu/ (accessed on 2 April 2025)) [47]. To further evaluate the predictive value of the CPS and CSS for immunotherapy response, we retrieved the GSE126044 dataset and the IMvigor210 cohort [48]. We included LUAD patients from GSE126044 and epithelial-origin patients with locally advanced or metastatic urothelial carcinoma receiving atezolizumab from IMvigor210. Patients were classified into two groups based on treatment response: complete response (CR)/partial response (PR) and stable disease (SD)/progressive disease (PD).

#### 4.2.6. Correlation Analysis Between ECPSs and Drug Sensitivity

To explore the association of the CPS and CSS with drug sensitivity in LUAD, half-maximal inhibitory concentration (IC_50_) data for 367 drugs were obtained from the Genomics of Drug Sensitivity in Cancer (GDSC) database (https://depmap.sanger.ac.uk/documentation/gdsc/ (accessed on 25 January 2026)) [49]. The oncoPredict package (v1.2) was used to predict IC_50_ values for TCGA-LUAD samples. Spearman’s rank correlation analysis was performed to evaluate the associations between CPS/CSS scores and the IC_50_ values of individual drugs. Drugs with significant opposite correlation patterns with the CPS and CSS were selected at adjusted *p* < 0.05 and |Cor| > 0.3. Pearson correlation analysis was performed to assess the associations between CPS/CSS scores and gene expression levels (adjusted *p* < 0.05, |R| > 0.3). Genes significantly positively correlated with drug IC_50_ (adjusted *p* < 0.05, R > 0.3) were defined as drug-resistance genes. Core overlapping genes underwent GO-BP enrichment analysis (adjusted *p* < 0.05) using clusterProfiler, and enriched terms were visualized as a semantic similarity network via the enrichplot package.

#### 4.2.7. Pan-Cancer Applicability of the CPS and CSS

To evaluate the pan-cancer applicability of the CPS and CSS, transcriptomic and clinical data for nine epithelial-cell-derived cancers were collected from the TCGA database: breast invasive carcinoma (BRCA), cholangiocarcinoma (CHOL), colon adenocarcinoma (COAD), esophageal carcinoma (ESCA), kidney renal papillary cell carcinoma (KIRP), liver hepatocellular carcinoma (LIHC), lung squamous cell carcinoma (LUSC), stomach adenocarcinoma (STAD), and thyroid carcinoma (THCA). CPS and CSS scores were calculated using ssGSEA (v1.52.3), and their diagnostic performance was evaluated by AUC across cancer types.

### 4.3. Statistical Analysis

Statistical analyses were performed using R software (v4.4.2) with Wilcoxon rank-sum, Fisher’s exact, and log-rank tests. For single comparisons, significance was set at *p* < 0.05; for multiple testing, Benjamini–Hochberg FDR correction was applied, and adjusted *p* < 0.05 was considered significant.

## 5. Conclusions

In summary, our study identified two ECPSs: the CPS and CSS. These two signatures displayed opposing expression patterns and biological functions in LUAD and could precisely characterize the dynamic epithelial-cell remodeling that occurs during malignant progression of LUAD. Multi-omics analyses further validated the utility of the CPS and CSS in diagnostic evaluation and prognostic stratification of LUAD. Moreover, we identified significant associations of the CPS and CSS with genomic mutation profiles, the tumor immune microenvironment, and drug sensitivity. Finally, *HMGA1*, a key gene in the CPS, was identified as a gene significantly associated with malignant progression in LUAD, suggesting its potential role as a candidate biomarker that merits further functional exploration.

## Data Availability

All the datasets can be downloaded directly from the indicated websites.

## Supplementary Materials

The following supporting information can be downloaded at: https://www.mdpi.com/article/10.3390/ijms27156819/s1.

## Abbreviations

The following abbreviations are used in this manuscript:

ECPSs	Epithelial-Cell Progression Signatures
LUAD	Lung adenocarcinoma
CPS	Cancer-Promoting Signature
CSS	Cancer-Suppressing Signature
TME	Tumor microenvironment
scRNA-seq	Single-cell RNA sequencing
ITH	Intratumor heterogeneity
KACs	KRT8+ alveolar intermediate cells
AIS	Adenocarcinoma in situ
MIA	Minimally invasive adenocarcinoma
IAC	Invasive adenocarcinoma
GEO	Gene Expression Omnibus
TCGA	The Cancer Genome Atlas
HB	Hemoglobin gene
HVGs	Highly variable genes
HR	Hazard ratio
ssGSEA	Single-sample gene-set enrichment analysis
HE	Hematoxylin–eosin
ROC	Receiver operating characteristic
AUC	Area under the curve
OS	Overall survival
TMB	Tumor mutation burden
TIDE	Tumor Immune Dysfunction and Rejection
CR	Complete response
PR	Partial response
SD	Stable disease
PD	Progressive disease
IC_50_	The half-maximal inhibitory concentration
GDSC	Genomics of Drug Sensitivity in Cancer
BRCA	Breast invasive carcinoma
CHOL	Cholangiocarcinoma
COAD	Colon adenocarcinoma
ESCA	Esophageal carcinoma
KIRP	Kidney renal papillary cell carcinoma
LIHC	Liver hepatocellular carcinoma
LUSC	Lung squamous cell carcinoma
STAD	Stomach adenocarcinoma
THCA	Thyroid carcinoma
NSCLC	Non-small cell lung cancer
