# Supplementary material for: Identifying Epithelial-Cell Progression Signatures (ECPSs) from Multi-Resolution Multi-Omics Data for Translational Clinical Applications in Lung Adenocarcinoma
Source: Int J Mol Sci. 2026 Jul 29;27(15):6819. doi: 10.3390/ijms27156819 (PMC13466838; doi:10.3390/ijms27156819)
Supplement: Supplementary file 1 [file ijms-27-06819-s001.zip › ijms-4321521-supplementary.pdf]

SUPPLEMENTAL METERIALS

Identifying Epithelial-Cell Progression Signatures (ECPSs) from Multi-Resolution Multi-Omics Data for Translational Clinical Applications in Lung Adenocarcinoma

Xueyao Chen, Tongxin Lv, Yang Wu, Fangfang Fan, Shaobo Kang, Renjie Dou, Wanmei Zhang, Dongxue Li, Rui Li, Yanyan Ping

College of Bioinformatics Science and Technology, Harbin Medical University, Harbin 150081, China.

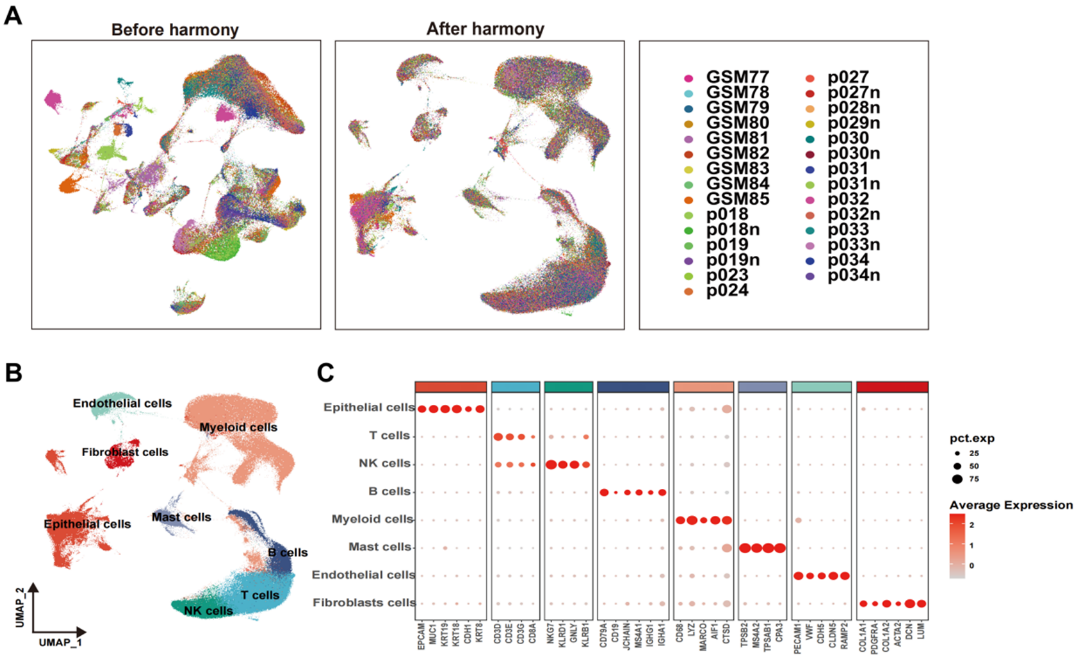

Supplementary Fig. S1. Quality control and cell annotation of scRNA-seq Data. (**A**) Batch effect correction of scRNA-seq data. (**B**) Cell type annotation of all cell clusters. C. Expression levels of marker.

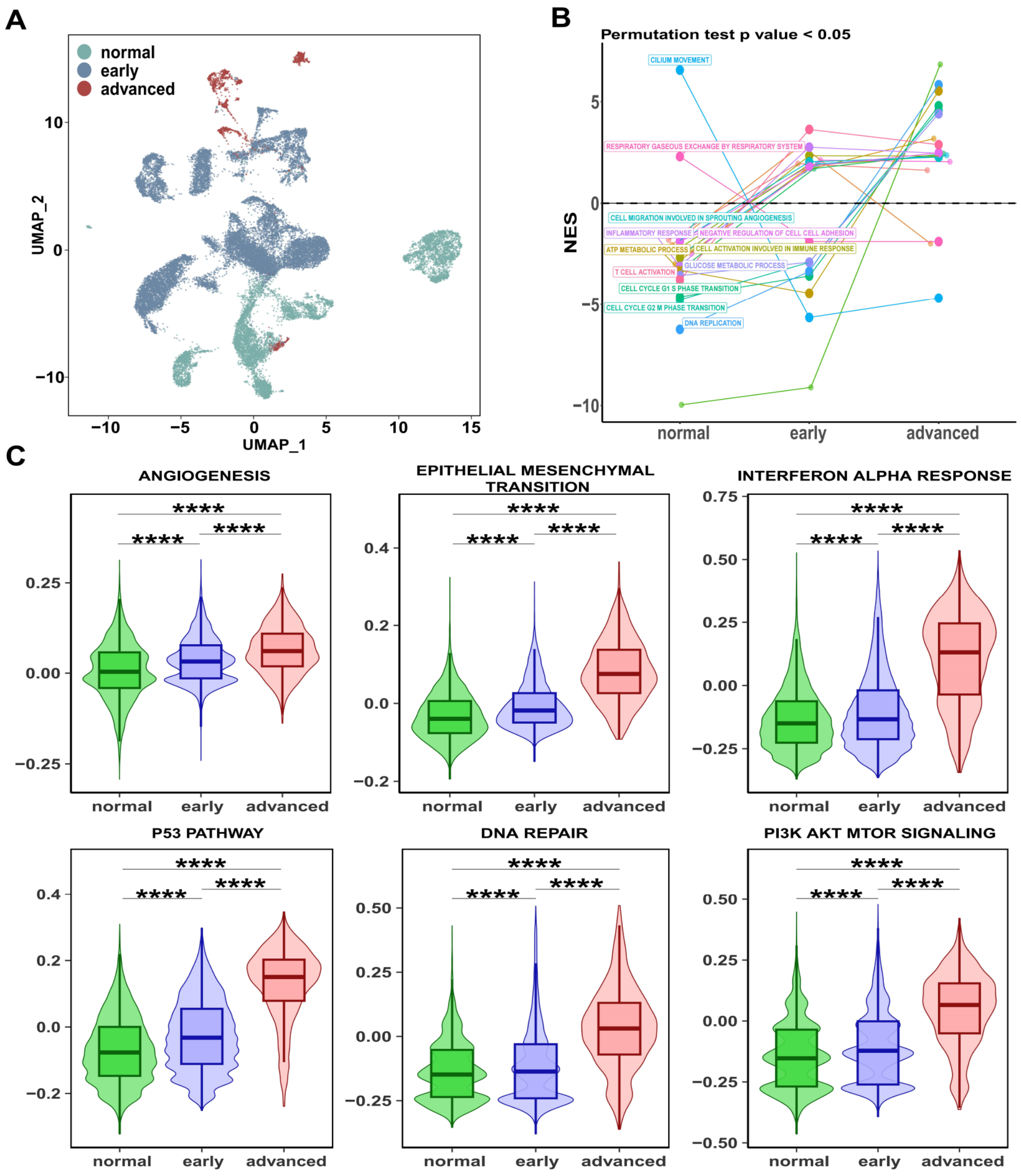

Supplementary Fig. S2. Epithelial cell lineage remodeling during LUAD progression. (**A**) UMAP plot of epithelial cells among normal, early-stage, and advanced-stage LUAD samples. (**B**) GO enrichment analysis of pathways related to normal lung function and cancer-causing pathways. (**C**) Differential activity of six cancer hallmark pathways calculated by ssGSEA.

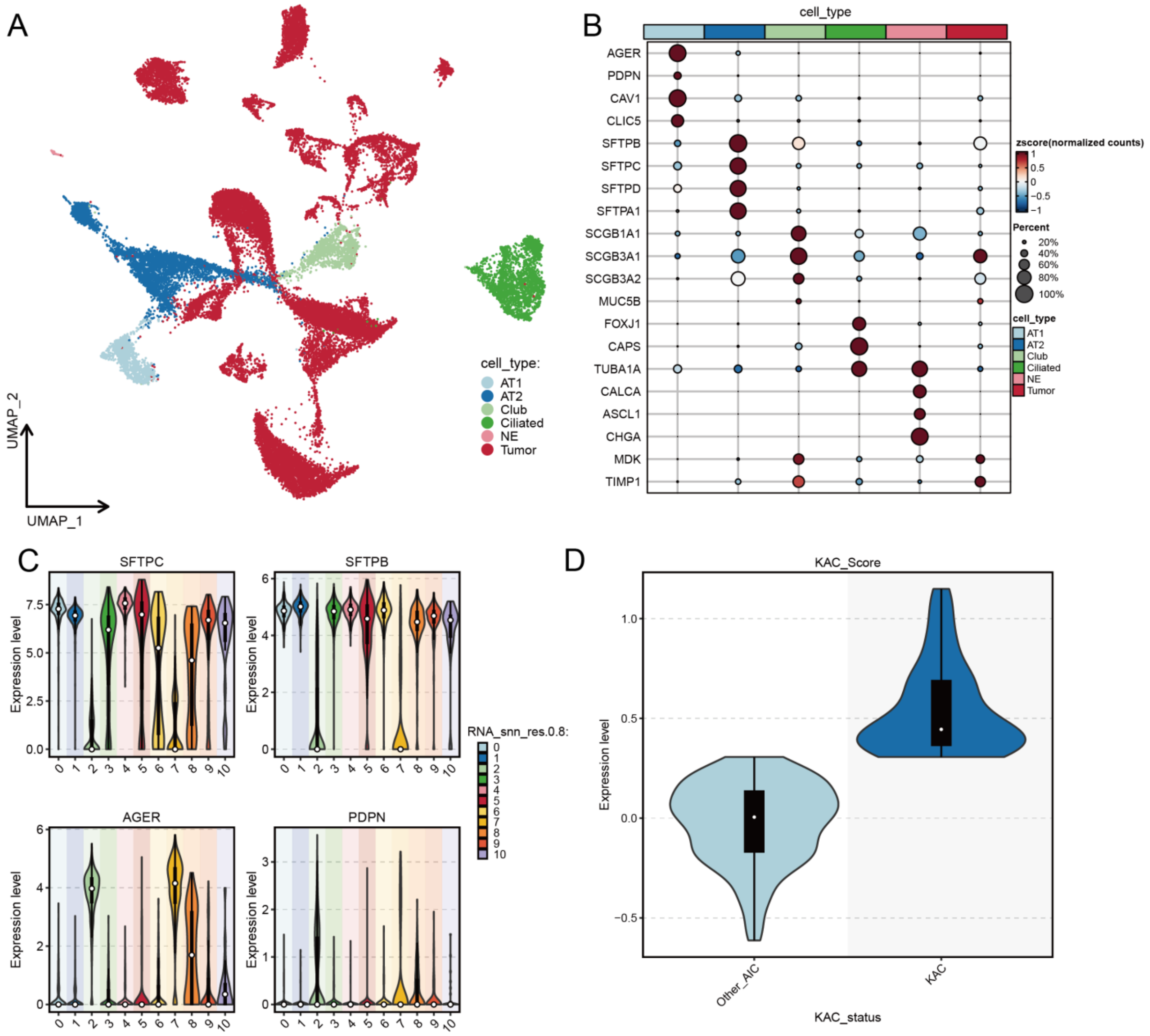

Supplementary Fig. S3. Cell type annotation of epithelial cells from scRNA-seq data. (**A**) UMAP visualization of epithelial cells colored by cell type, including AT1, AT2, Club, ciliated, NE, and tumor cells. (**B**) Dot plot showing the expression of canonical cell-type marker genes across clusters. (**C**) Violin plots showing the expression distribution of representative marker genes in each cluster. (**D**) Violin plot comparing KAC scores between KAC cells and other epithelial cells.

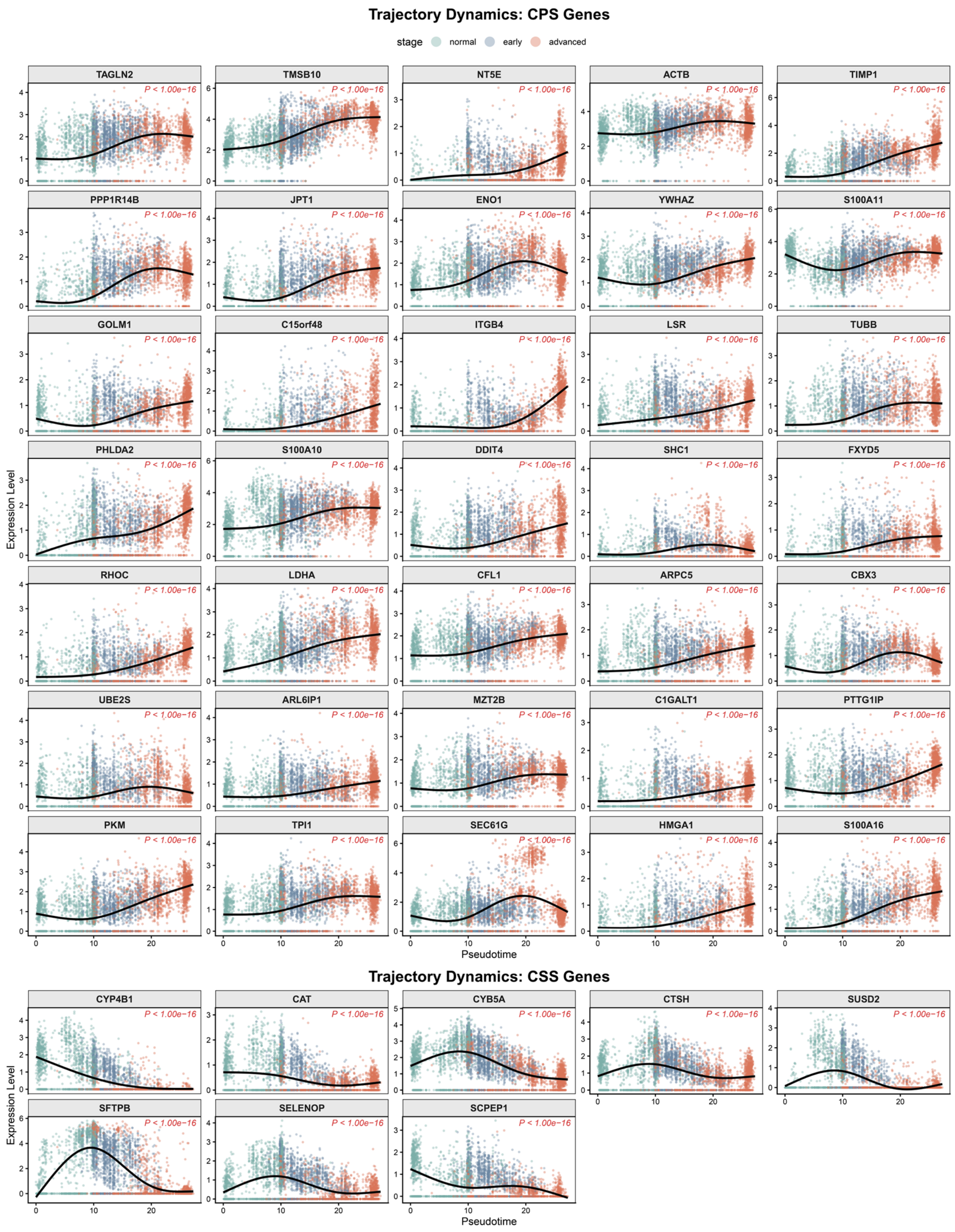

Supplementary Fig. S4. Dynamic changes of CPS/CSS genes along pseudotime.

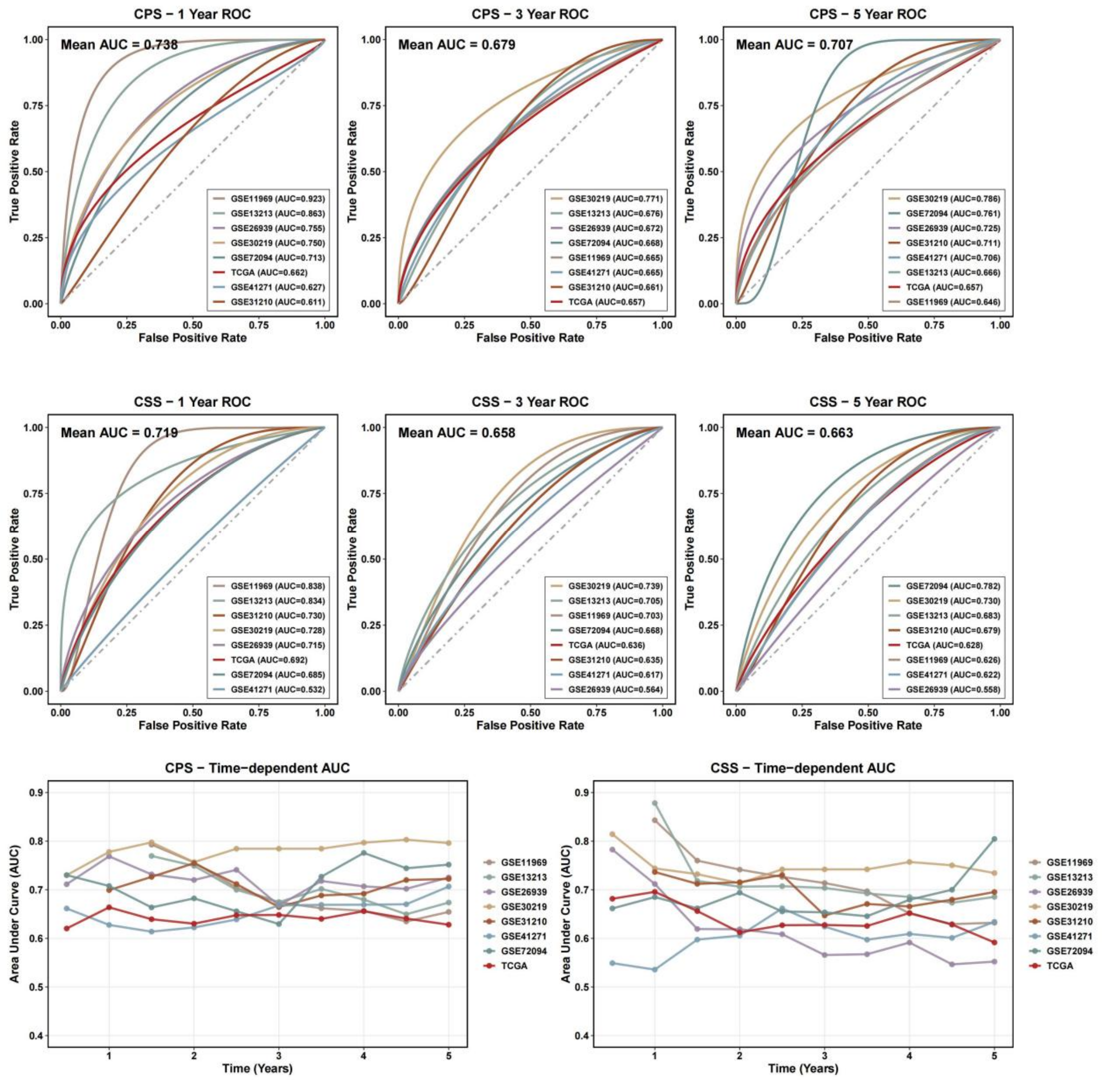

Supplementary Fig. S5. ROC curves of CPS and CSS for survival prediction across eight datasets.

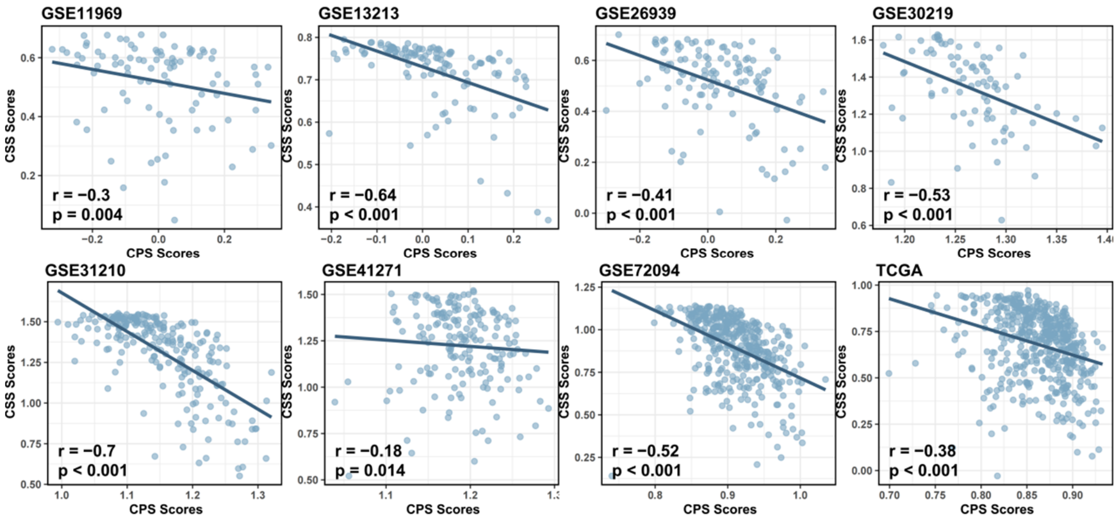

Supplementary Fig. S6. Correlation between CPS and CSS in eight bulk sequencing Datasets.

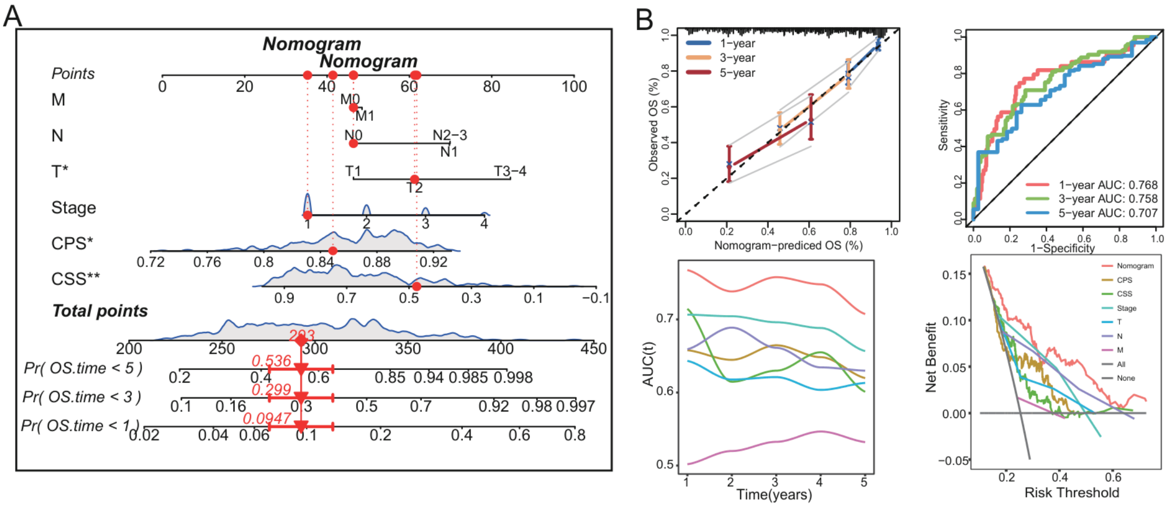

Supplementary Fig. S7. Prognostic nomogram integrating CPS, CSS, and clinicopathological features for survival prediction in LUAD. (**A**) Nomogram integrating CPS, CSS and clinicopathological features for 1-, 3-, and 5-year OS prediction. (**B**) Calibration plots and time-dependent ROC curves evaluating the accuracy of the nomogram. Time-dependent AUC curves and DCA comparing the nomogram against individual clinical factors.

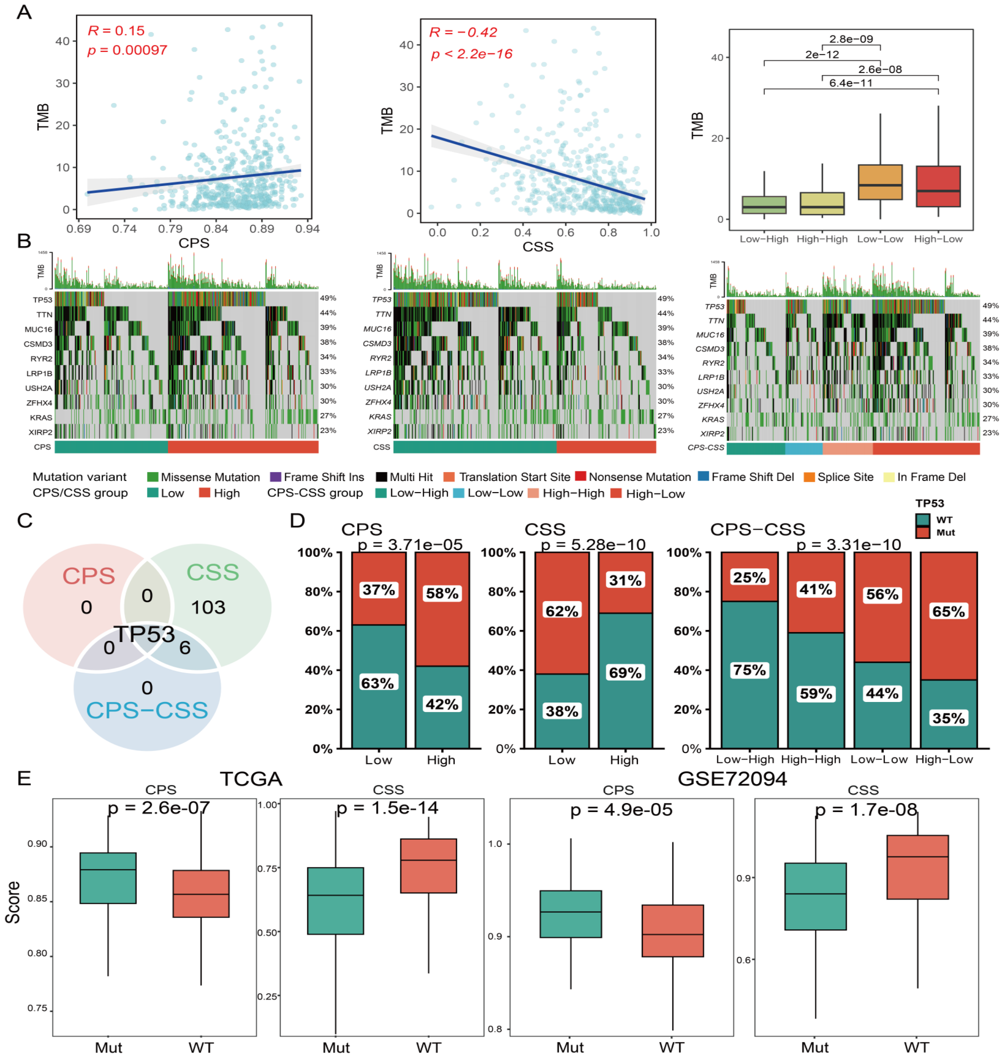

Supplementary Fig. S8. Associations of CPS/CSS with TMB and genomic instability in LUAD. (**A**) Correlation analyses of CPS/CSS with TMB, and comparison of TMB levels across four groups (**B-C**) Mutation frequencies of common mutated genes in different CPS/CSS groups. (**D**) TP53 mutation frequency across different CPS/CSS groups. (**E**) Differences in CPS/CSS scores between TP53-mutant and TP53-wild type tumors in TCGA-LUAD and GSE72094 cohorts.

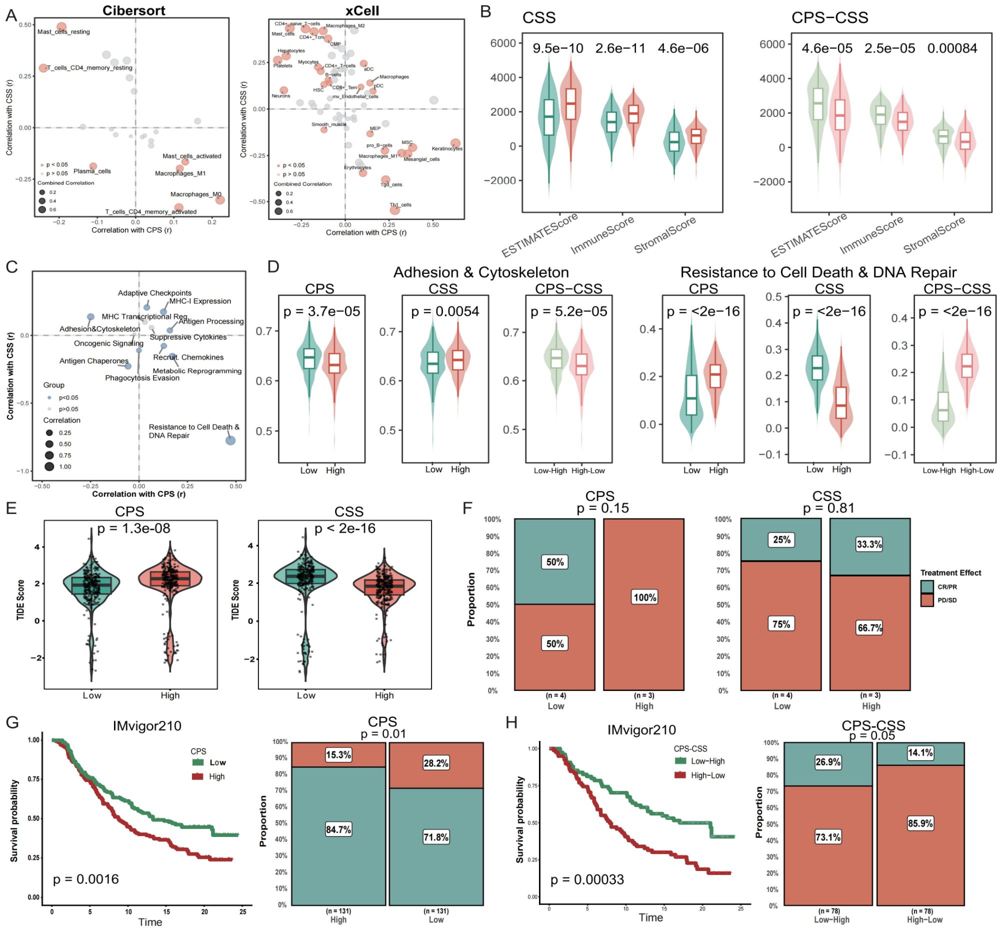

Supplementary Fig. S9. Associations of CPS/CSS with tumor microenvironment, immune escape, and immunotherapy response. (**A**) Correlation analyses of CPS/CSS scores with TME cellular proportions. (**B**) Comparison of ESTIMATE, Immune, and Stromal scores among different CSS groups and CPS-CSS combined groups. (**C-D**) Correlations between immune escape CPS_{high} pathways and CPS/CSS scores. (**E**) Comparison of TIDE scores in different CPS/CSS groups in TCGA-LUAD. (**F**) Differences in immunotherapy response rates (CR/PR) between different CPS/CSS groups in the GSE126044 cohort. (**G**) Differences in immunotherapy response rates (CR/PR) and OS between CPS_{high} and CPS_{low} groups in IMvigor210 cohort. (**H**) Differences in immunotherapy response rates (CR/PR) and OS between CPS_{low}-CSS_{high} and CPS_{high}-CSS_{low} groups in IMvigor210 cohort.

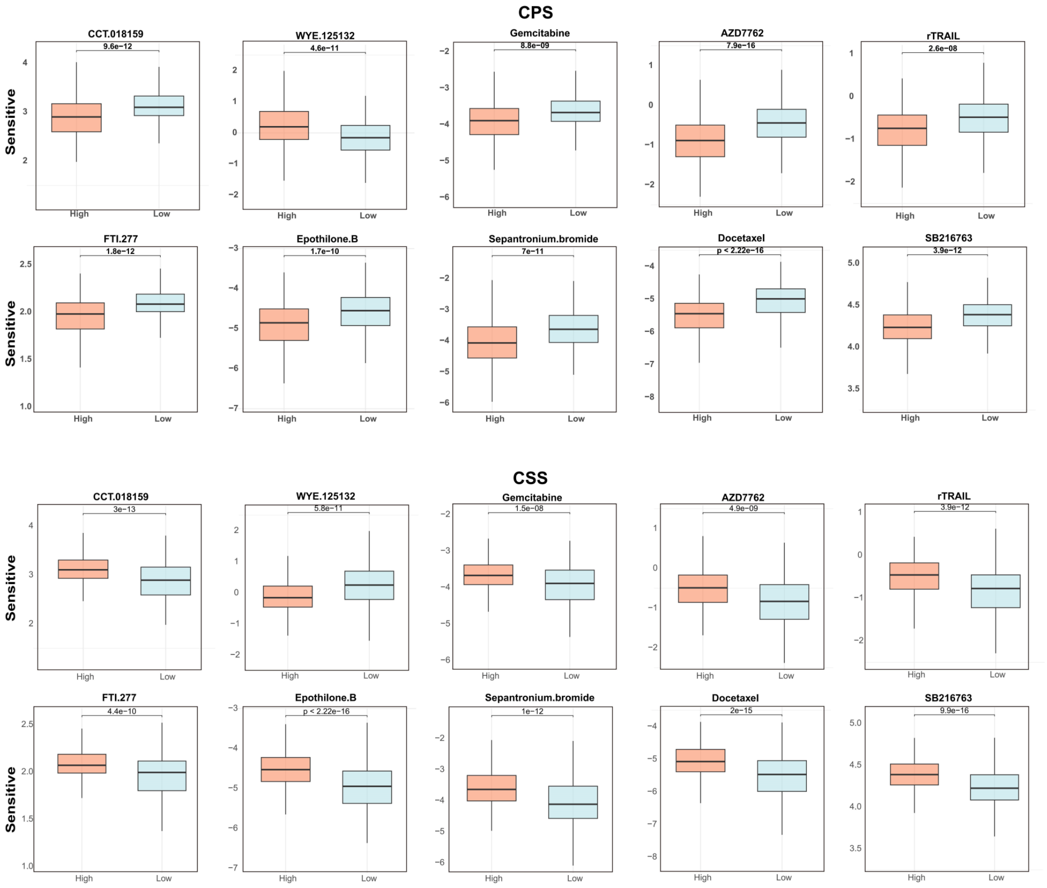

Supplementary Fig. S10. Comparison of anticancer drugs sensitivity in different CPS and CSS combination groups.

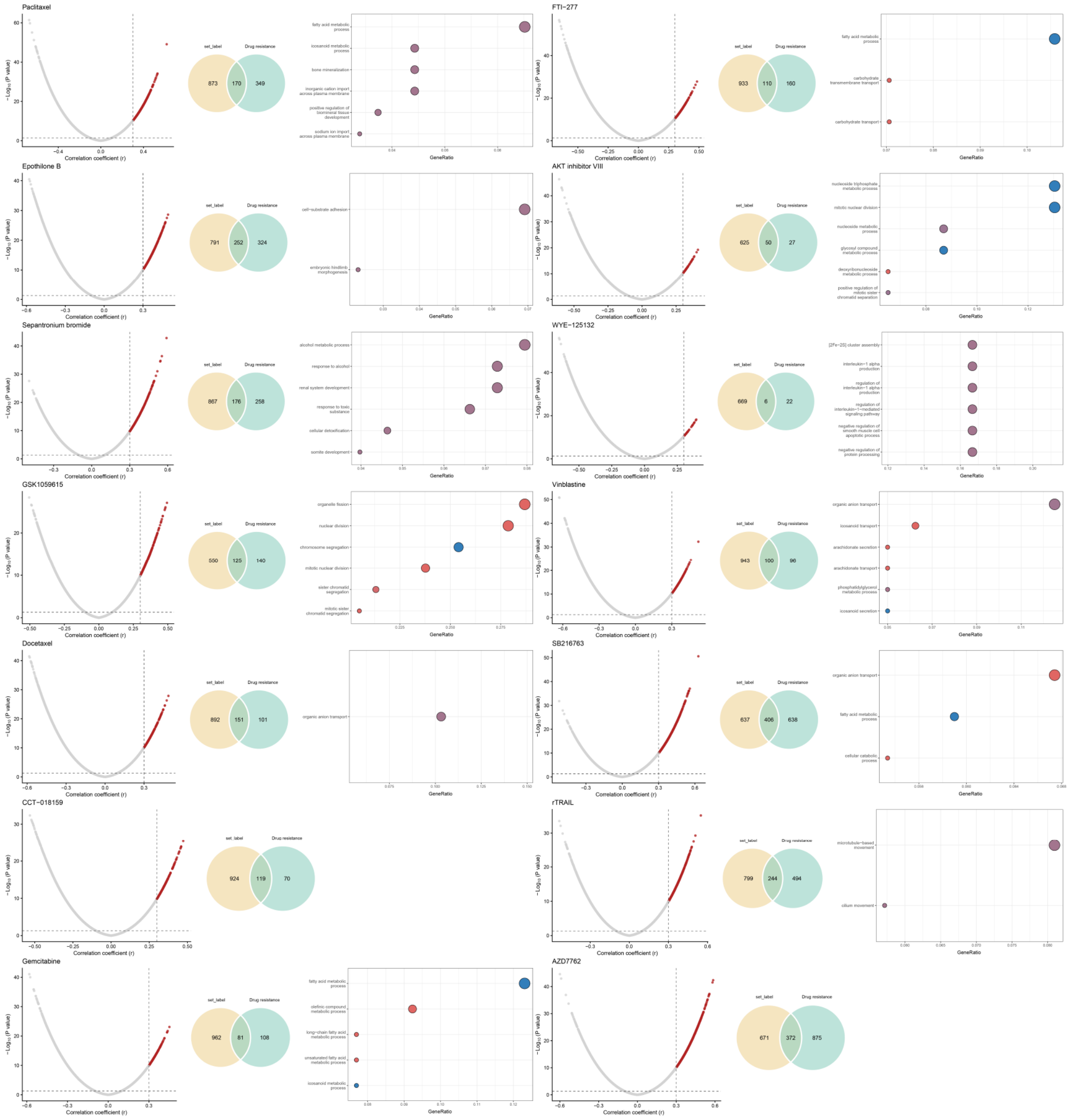

Supplementary Fig. S11. Identification of CPS/CSS-associated drug resistance genes and GO-BP functional enrichment analysis across 14 drugs.

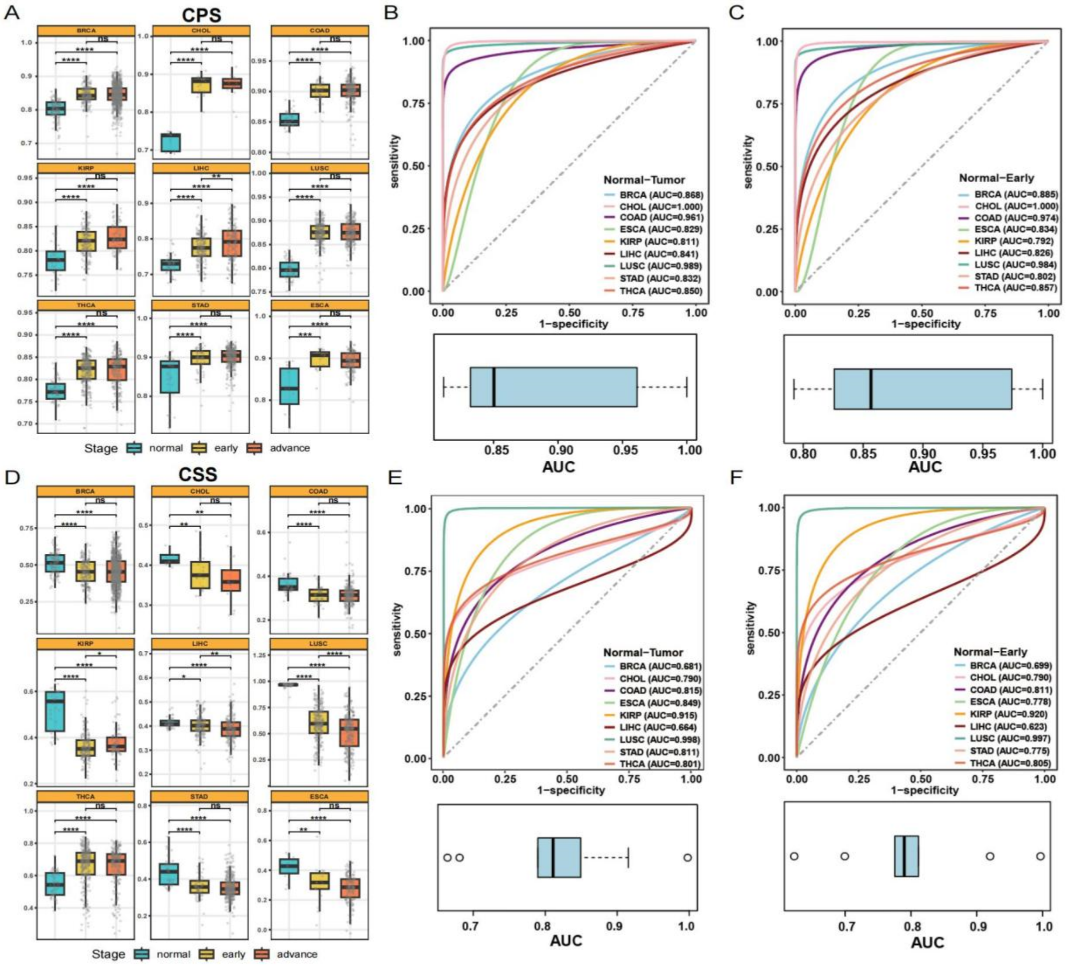

Supplementary Fig. S12. Pan-cancer applicability of CPS and CSS across epithelial malignancies. (**A**) Comparison of CPS scores across normal, early and advanced-stage in nine TCGA epithelial cohorts. (**B-C**) ROC curves and AUC distributions showing diagnostic performance of CPS in tumor or early-stage versus normal tissues across nine independent cohorts. (**D**) Comparison of CSS scores across normal, early and advanced-stage in the same cohorts. (**E-F**) ROC curves and AUC distributions showing diagnostic performance of CSS in tumor or early-stage versus normal tissues across nine independent cohorts.

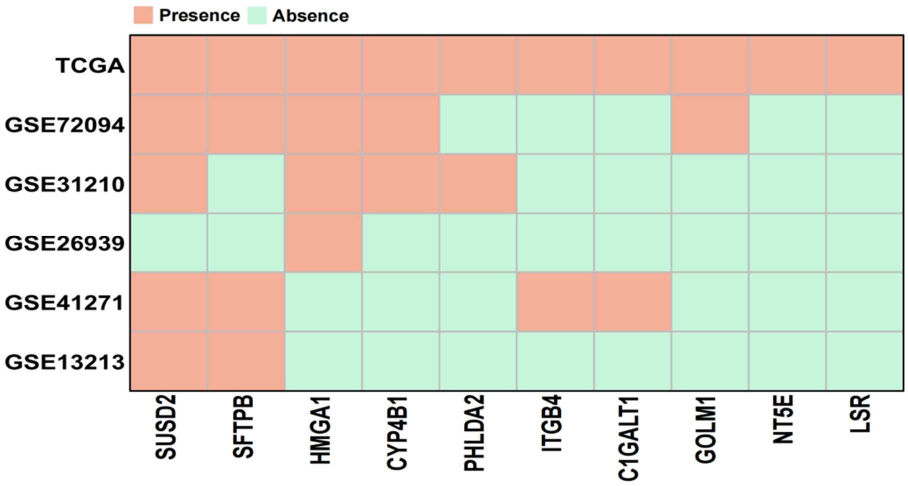

Supplementary Fig. S13. Prognostic association of epithelium-specific expression genes in six bulk sequencing datasets.

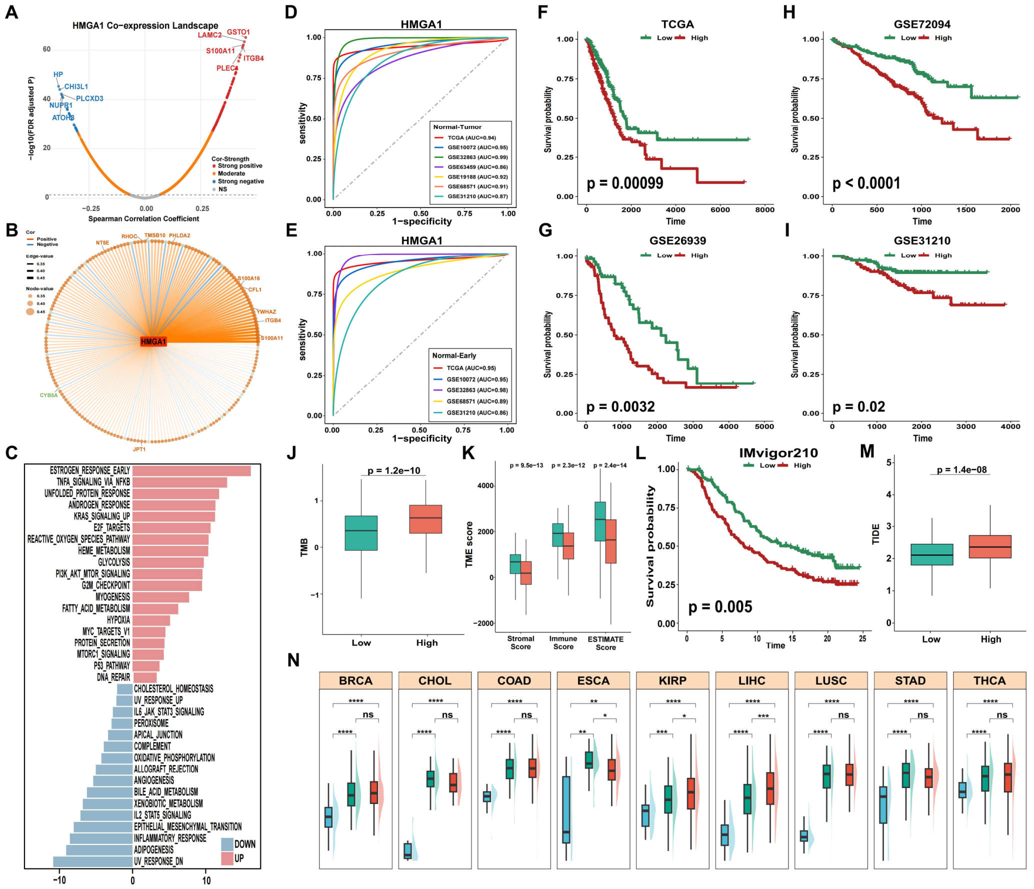

Supplementary Fig. S14. Functional characterization of HMGA1 in LUAD progression. (**A-B**) Co-expressed network of HMGA1 in advanced-stage epithelial cells. (**C**) Enrichment analysis of HMGA1 co-expressed genes. (**D-I**) Diagnostic and prognostic performance of HMGA1 in LUAD. (**J**) Comparison of TMB in high- and low-HMGA1 expression groups in TCGA-LUAD cohort. (**K**) Comparison of ESTIMATE, Immune, and Stromal scores in high- and low-HMGA1 expression groups. (**L-M**) Differences in TIDE scores and OS between high- and low-HMGA1 expression groups (IMvigor210). (**N**) HMGA1 expression levels in tumor and normal tissues across nine epithelial malignancies.

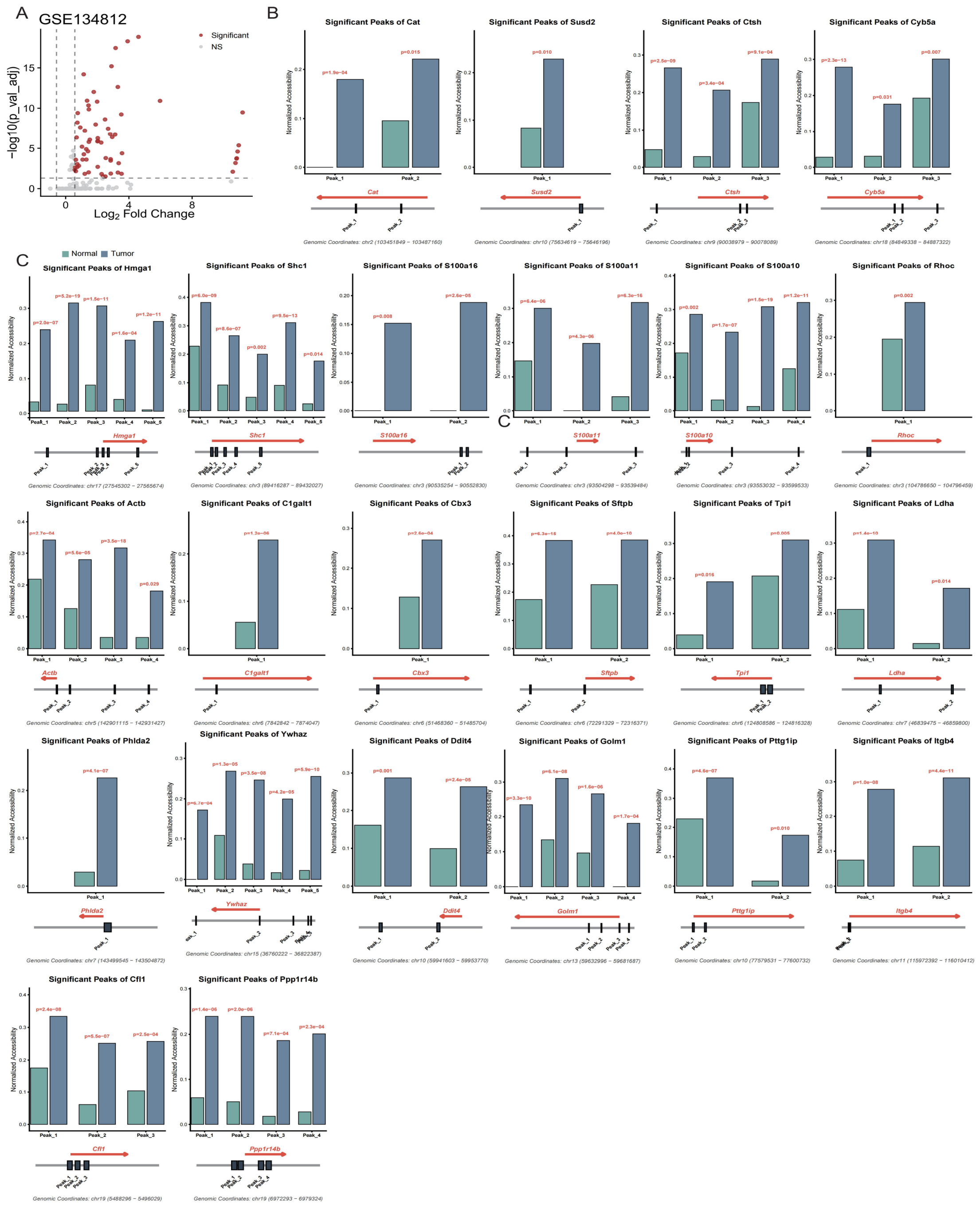

Supplementary Fig. S15. Chromatin accessibility alterations of CPS/CSS genes in LUAD. (**A**) Volcano plot of differentially accessible peaks from the GSE134812 scATAC-seq dataset. (**B**) Chromatin accessibility of CSS gene regulatory regions in the GSE134812 scATAC-seq dataset. (**C**) Chromatin accessibility of CPS gene regulatory regions in the GSE134812 scATAC-seq dataset.

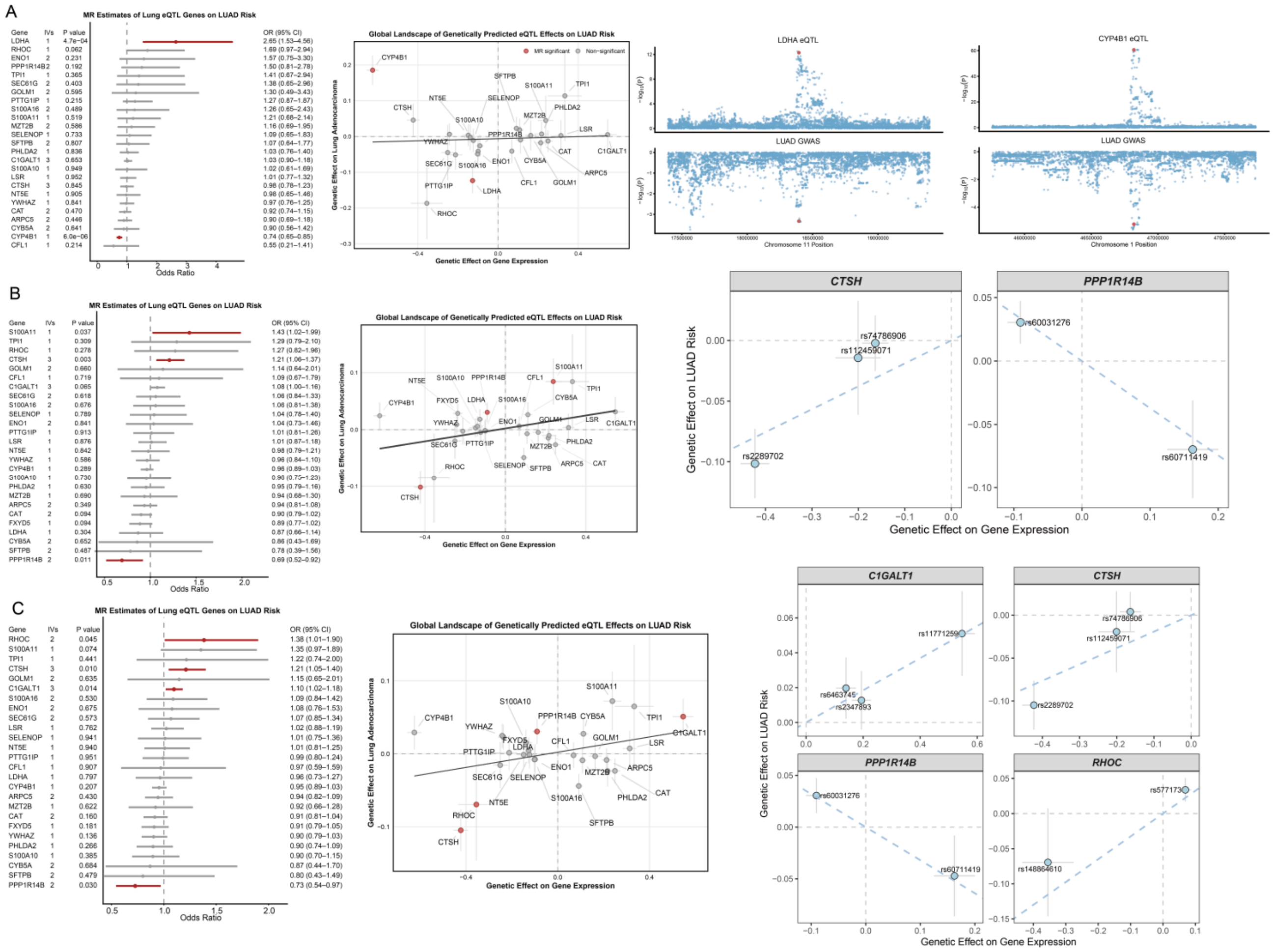

Supplementary Fig. S16. Two-sample Mendelian randomization analysis of CPS/CSS genes and LUAD risk.

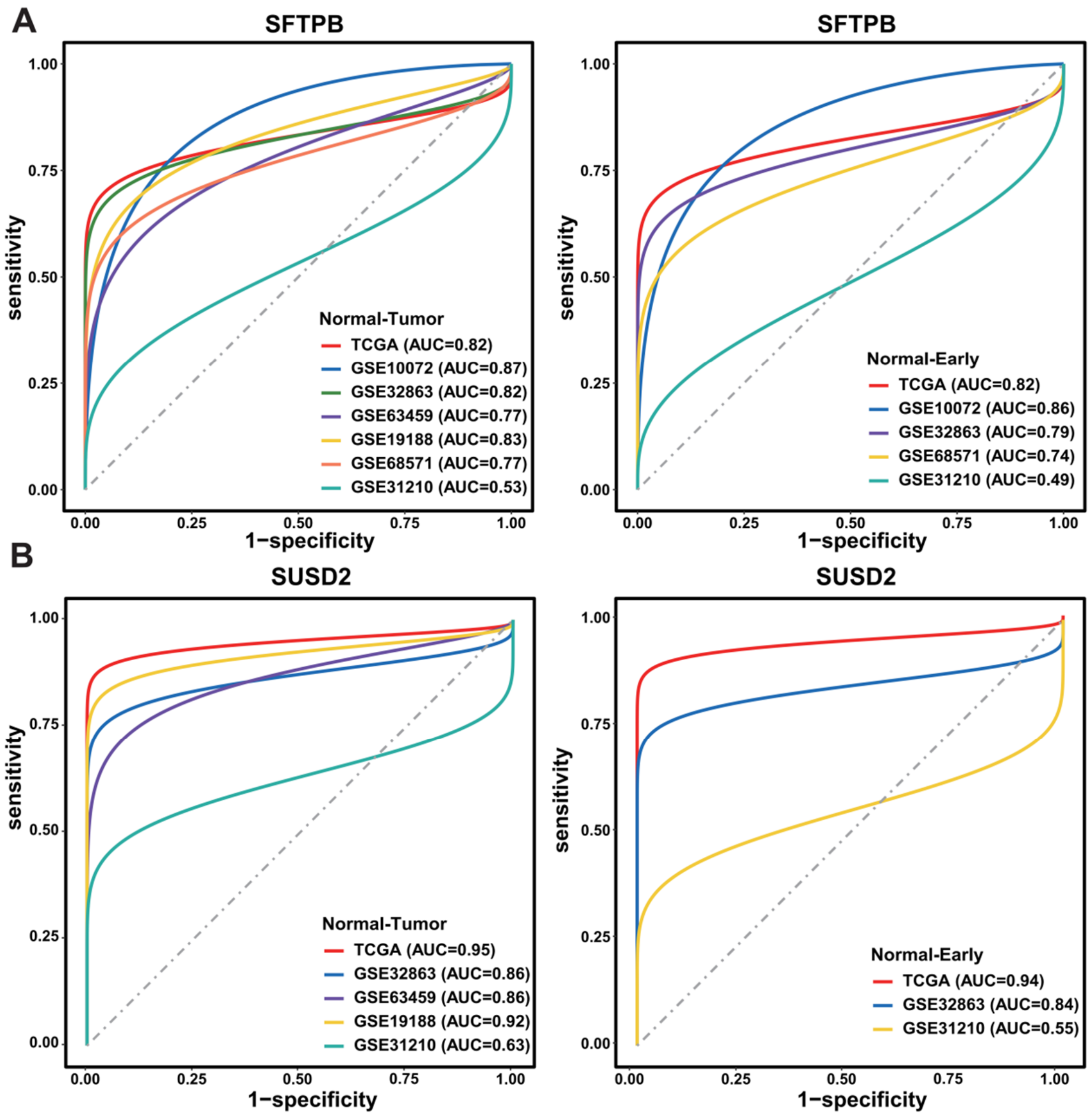

Supplementary Fig. S17. Diagnostic efficacy of SFTPB and SUSD2 gene in LUAD. (**A**) ROC curves of SFTPB diagnostic performance in LUAD tumor or early-stage versus normal tissues across seven independent cohorts. (**B**) ROC curves of SUSD2 diagnostic performance in LUAD tumor or early-stage versus normal tissues.

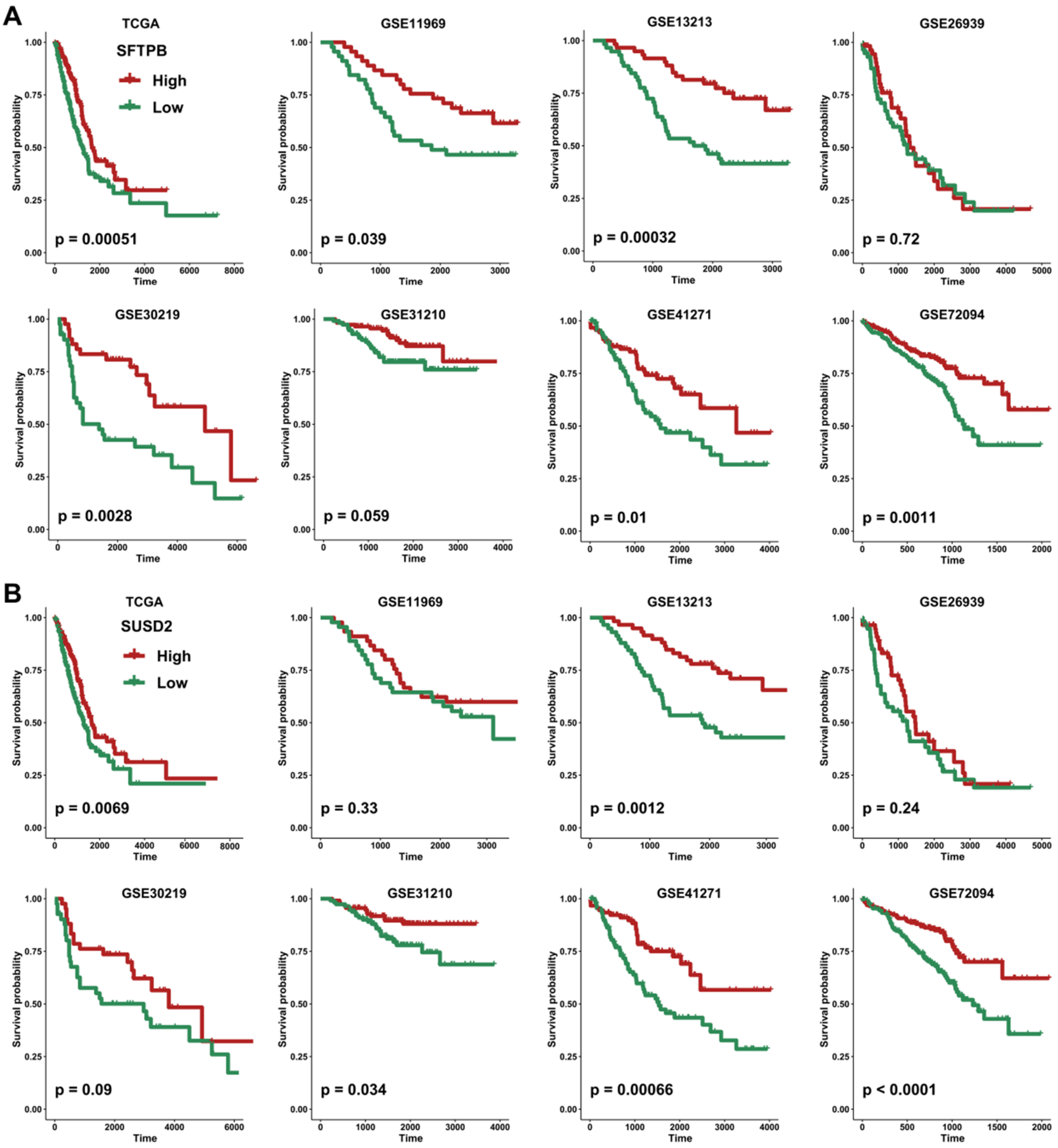

Supplementary Fig. S18. Prognostic value of SFTPB and SUSD2 gene in LUAD. (**A**) Survival analysis of patients stratified by SFTPB expression in eight bulk cohort. (**B**) Survival analysis of patients stratified by SUSD2 expression in eight bulk cohort.

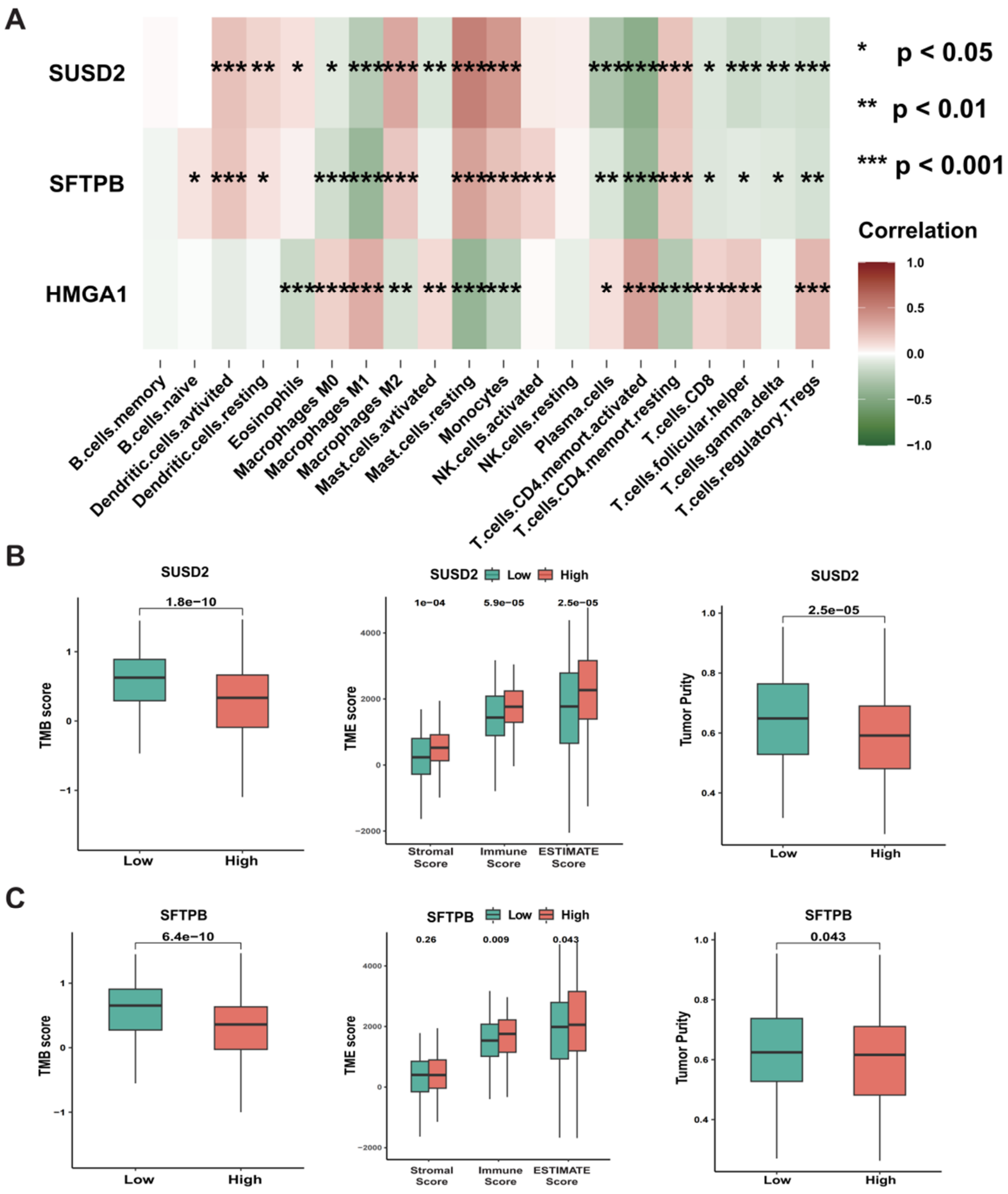

Supplementary Fig. S19. Correlation between SFTPB and SUSD2 genes and immune microenvironment and TMB in LUAD. (**A**) Correlation between SFTPB, SUSD2, HMGA1 and the proportion of immune cells. (**B**) Differences in TMB score, immune infiltration score, and tumor purity between high and low expression groups of SUSD2. (**C**) Differences in TMB score, immune infiltration score, and tumor purity between high and low expression groups of SFTPB.

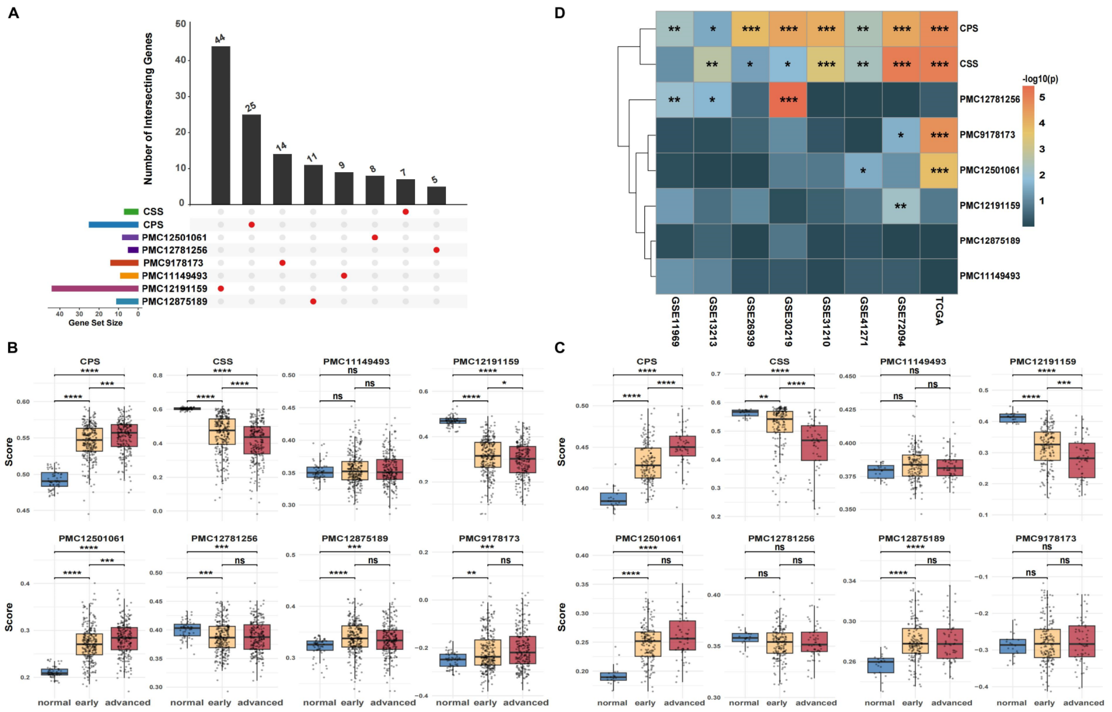

Supplementary Fig. S20. Comparison of CPS and CSS with publicly available epithelial progression signatures in LUAD. (**A**) The gene overlap between CPS/CSS and six previously published LUAD progression-associated signatures. (**B-C**) The distribution of signature scores across different tumor stages in the TCGA-LUAD and GSE31210 cohort. (**D**) The prognostic performance of each signature across eight independent cohorts.
